# Hydrodynamic activities and lifestyle preferences synergistically drive prokaryotic community assembly processes in the dual fronts system of the Yangtze River Estuary

**DOI:** 10.3389/fmicb.2025.1610617

**Published:** 2025-07-31

**Authors:** Han Lu, Zhonglin Ma, Lei Su, Yunfei Du, Kun Zhou, Peng Wang

**Affiliations:** ^1^State Key Laboratory of Marine Geology, Tongji University, Shanghai, China; ^2^School of Ocean and Earth Science, Tongji University, Shanghai, China

**Keywords:** Yangtze River (Changjiang River), dual fronts, hydrodynamic, particle-associated (PA), free-living (FL), lifestyle preferences, assembly process

## Abstract

The dual fronts system of the Yangtze River Estuary plays a critical role in the hydrodynamic-biological coupling mechanisms, whose frontal effects stimulate marine microorganisms to adapt to environmental fluctuation. However, the synergistic mechanisms driving prokaryotic community assembly in the dual fronts system remain poorly conceptualized, particularly regarding lifestyle preferences (free-living vs. particle-associated). By integrating physicochemical parameter analysis and high-throughput 16S rRNA amplicon sequencing, we described the unique prokaryotic community and quantified the assembly processes of particle-associated and free-living prokaryotic sub-communities. The effects of the dual fronts reshaped the prokaryotic community by differentiating the abundant and rare species of the particle-associated and free-living communities. *Rhodobacteraceae, Flavobacteriaceae*, and *Cyanobiaceae* played vital roles in the prokaryotic community across three water masses, while the rare species exhibited distinct differences. The prokaryotes in the water mass between the sediment front and plume front preferred a particle-associated lifestyle, while free-living was the preferred lifestyle in other water masses. Stochastic dispersal limitation and deterministic homogeneous selection dominated prokaryotic community assembly in the dual fronts system. Free-living prokaryotes with high environmental sensitivity were influenced by homogeneous selection in community assembly, and particle-associated prokaryotes were easily constrained by particle-mediated dispersal. The vigorous hydrodynamic activities could stimulate the attachment-detachment on particulates of prokaryotes, resulting in alterations to assembly mechanisms and participative species. Ultimately, hydrological activities and lifestyle preferences collaborated to determine the assembly mechanisms of meta-community and sub-community. This study pioneers the linkage between dual frontal hydrodynamics and microbial lifestyle-specific assembly, providing a predictive framework for prokaryotic community responses under tumultuous environmental fluctuations.

## 1 Introduction

Ocean fronts are important (sub-)mesoscale physical processes that are widely spread in the ocean environments, including estuary, continental shelf, and open sea (McWilliams, 2021). The environmental gradients on each side of the fronts compartmentalize extensive water regions into distinct water masses at mesoscale (10–100 km) and sub-mesoscale (1–10 km) spatial scales (Liu et al., 2018; Cai et al., 2022; Xing et al., 2023a,b, 2025). Meanwhile, the frontal zones exhibit pronounced and discontinuous hydrodynamic characteristics (Hopkins et al., 2010; McWilliams, 2021), stimulate microbial community responses (Phoma et al., 2018; Cai et al., 2022), and delineate the biogeographic boundaries of plankton combinations (Baltar et al., 2016; Liu et al., 2018; Lemonnier et al., 2020). Consequently, combining microbial community adaptation to environmental gradients with hydrological activities is essential to reveal the bioecological consequences resulting from the frontal effects.

With the advancement of null model analysis, current marine microbial ecology research marks a novel emphasis on the community assembly processes, indicating the mechanisms of forming microbial biodiversity and maintaining the community stability. Both deterministic processes correlating with natural selection and stochastic processes driven by random events are the prevailing mechanisms of community assembly (Chen et al., 2019; Jiao et al., 2020; Sun et al., 2022). Assembly mechanisms preferentially play roles in the aggregation and dispersal of sub-community at a more precise biological organization level rather than the whole meta-community, such as particular driving species and genotypes (Ning et al., 2020). For example, the environmental selection enhances metabolism by sorting species, which can eliminate specific taxa metabolizing scarce resource pools and impair community metabolic functioning (Graham et al., 2016; Ning et al., 2020). Similarly, the ecological selection can encourage a particular species to assemble within a single microbial community, while other species still experience a predominant assembly driven by stochastic processes (Ning et al., 2020; Guo et al., 2020). However, previous studies have mainly concentrated on the overall meta-community assembly lacking conceptualization on core sub-community preferentially experiencing the assembly processes. More importantly, previous studies emphasized the determinants of the biotic factors (e.g., interspecific competition), abiotic factors (environmental variations), and random events (e.g., dispersal) on the microbial community assembly processes. The lifestyle preferences induced by niche filtering can serve as a crucial biotic factor among the factors mentioned above, exhibiting as the free-living (FL) and particle-associated (PA) lifestyles for aquatic microorganisms (Logares et al., 2018; Li R. et al., 2021; Bachmann et al., 2018; Chun et al., 2021; Zhang et al., 2023). The particles provide the colonization habitat and available organic materials for PA microorganisms, while FL microorganisms benefit from the released organic matter by PA microorganisms in the surrounding microenvironment. The assembly processes in PA community are exclusively driven by stochasticity, while homogeneous selection accounts for a 30% contribution to FL community assembly in the Yangtze River Estuary (Shi et al., 2022). Furthermore, the highly variable dynamic characteristics hinder the monitoring of abiotic factors and complicate the deciphering of microbial response to multiple environmental variations in the frontal zone. It is the hydrodynamic activity that determines environmental changes. Therefore, hydrodynamic activity can function as a more decisive factor than niche filtering to community assembly, such as the passive diffusion in surface water (Villarino et al., 2018; Hou et al., 2024). For example, the cascade dams located along the Yangtze River mainstream can disturb the pre-existing hydrological conditions in the estuarine and adjacent waters. Eventually, this disturbance regulates the suspended particulate concentrations and shifts the microbial functionality and community assembly (Gao et al., 2021).

The Yangtze River Estuary (Changjiang River Estuary, CRE) is located at the intersection of river, land, and ocean environments with a complicated hydrodynamic system. In summer, a typical sediment front (121.9°E–122.5°E, SF/on-SF) originates where the Changjiang Diluted Water (CDW) carrying loads of sediments collaborates with the tide-induced mixing in the CRE (Ge et al., 2020; Du et al., 2025). As CDW expands outwards, the high salinity continental shelf water finally meets with it and forms a plume front (122.5°E–123.5°E, PF/on-PF) at the outer CRE (Li W. et al., 2021). The cross-frontal exchange with adjacent water masses forms a transitional water mass between the SF and PF water masses (BE/between-SF-PF) with distinct ecological gradients (Bower and Lozier, 1994). Consequently, the variable location of the sediment front and plume front is determined by the salinity and total suspended matter gradients (Li W. et al., 2021; Tu et al., 2024; Du et al., 2025). The dual fronts system of the Yangtze River Estuary (Changjiang River Estuary Dual Fronts, CDF) functions as a natural experimental site to integrate prokaryotic ecological effects with marine physical dynamics (Du et al., 2023; Hou et al., 2024; Xu et al., 2025).

To prove the importance of the sediment front and plume front to delineate ecosystem boundaries, we collected seawater samples along the CDF system in the summer of 2023. Subsequently, we measured the environmental variations and conducted 16S rRNA gene amplicon sequencing to assess the correlations between environmental variations (turbidity, salinity, temperature, dissolved oxygen, pH and inorganic nutrients) and prokaryotic community, community structure diversity, lifestyle preferences, and community assembly mechanisms. We demonstrated the significant differences in unique prokaryotic communities across distinct water masses and emphasized homogeneous selection and dispersal limitation synergistically governed the community assembly. We additionally examined the interaction between hydrodynamical activities and lifestyle preferences in driving community assembly. Moreover, we underscored the vital role of hydrodynamic-biological coupling in the dual fronts system, which will enhance our understanding of the bioecological consequences of the frontal effects and plankton dynamics in response to environmental fluctuations.

## 2 Methods

### 2.1 Sample collection and physicochemical characteristics of water mass

**Figure 1:**
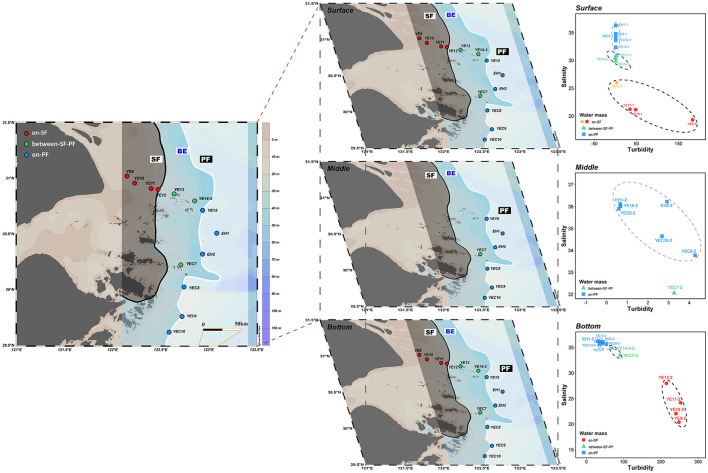
An overview of the study area and sampling stations. The location of dual fronts was observed derived by a previous study (Li W. et al., 2021). The water masses in surface, middle, and bottom water were clustered by salinity, turbidity, and other optimal environmental parameters. A black shadow with a red spot covering the SF area; light blue shadow with a green spot covering the BE area; white shadow with a blue spot covering the PF area, solid black line: SF location; solid white line: PF location. The map was generated with Ocean Data View (Reiner, 2024).

Seawater samples were collected during the Bohaike Research Vessel cruise from August 17 to 22 in 2023, which investigated the waterways extending outward from the southern CRE trough to the East China Sea shelf (122.15°E–123.08°E, 29.62°N–31.02°N, Supplementary Table S2). Based on the situation of SF (121.9°E–122.5°E) and PF (122.5°E–123.5°E) delineated from multi-year observational data in and off the CRE during the summers of 1988–2016 by JianZhong Ge (Li W. et al., 2021), we set up a total of thirteen stations across the CDF system (Supplementary Table S2). In the study area, four stations are set up on the nearshore water mass of SF, six stations on the offshore water masses of PF, and three stations between SF and PF. A total of 66 seawater samples were collected from three layers of the water columns (Figure 1). The surface samples were collected at a depth of 0–2 m beneath the sea surface. The samples collected at a depth of 3–4 m above the seabed were considered as the bottom samples. The seawater sample of the middle layer was taken only when the station depth exceeded 30 m. Briefly, all seawater samples were collected using a conductivity-temperature-depth (CTD) rosette sampler (Sea-Bird Electronics, SBE 32, USA) equipped with 8 L Niskin bottles. Meanwhile, in each depth profile, environmental parameters such as salinity, temperature, turbidity, pH and dissolved oxygen (DO) were monitored *in situ* using EXO3 Multiparameter Sonde (YSI, Yellow Springs, Ohio, USA). We identified the cluster patterns of the SF, BE, and PF water masses and reassembled the stations among the surface, middle, and bottom water layers (Figure 1), based on turbidity, salinity, and the other optimal environmental parameters (Supplementary Table S3). Dissolved inorganic nitrogen (DIN: $NO_{3}^{-}$ + $NO_{2}^{-}$ + $NH_{4}^{+}$), dissolved inorganic phosphate (DIP), and dissolved silicate (DSi) were analyzed using a SmartChem automated discrete chemistry analyzer (SmartChem200, Alliance, France) at the Third Institute of Oceanography, Ministry of Natural Resources (TIO). At each water layer, a total of 2 L of seawater was collected in duplicate and then filtered immediately for the collection of microbial cells. Filtration was performed sequentially through 3 μm and then 0.22 μm Millipore polycarbonate filters (Millipore Corporation, Billerica, MA, USA) to obtain PA and FL microorganisms for high-throughput sequencing, respectively.

### 2.2 DNA extraction, amplification and sequencing

To extract total microbial genomic DNA from both 3 μm and 0.22 μm filters, we utilized the DNeasy^Ⓡ^ PowerWater^Ⓡ^ kit (Qiagen, Germany) following the manufacturer instructions. The DNA quality was detected through 1.6% agarose gel electrophoresis, and its concentration was quantified using a NanoDrop2000 spectrophotometer (Thermo Scientific, United States). The amplified DNA library preparation was conducted using the NEXTFLEX Rapid DNA-Seq Kit for Illumina^Ⓡ^ (Bioo Scientific, USA) following the standard protocol. The ~390 base pairs (bp) in the hypervariable V4 region of the prokaryotic 16S rRNA gene were amplified with a universal primer pair 515F (5′-GTG YCA GCM GCC GCG GTA A-3′) and 806R (5′-GGA CTA CNV GGG TWT CTA AT-3′) by a T100 Thermal Cycler PCR thermocycler (BIO-RAD, USA). Each PCR reaction mixture was programmed to a 20 μL system, including 10 μL of 2 × Pro Taq Master Mix, 0.8 μL of each forward and reverse primer (5 μM), a volume containing 10 ng of template DNA, and nuclease-free water to a final volume of 20 μL. The PCR amplification cycling procedure was comprised of initial pre-denaturation at 94°C for 5 min followed by 27 cycles of denaturing at 95°C for 30 s, annealing at 55°C for 30 s, extension at 72°C for 45 s, single extension at 72°C for 10 min and end at 4°C. The PCR products were recovered using a 2% agarose gel and later purified using a DNA gel recovery kit (PCR Clean-Up Kit, China). The purified amplicons were pooled in equimolar concentrations and subjected to paired-end sequencing on a PE300 Illumina Nextseq2000 platform (Majorbio Bio-Pharm Technology Co. Ltd., Shanghai, China).

### 2.3 Bioinformatic analysis

The raw sequences data of 16S rRNA genes were processed using QIIME2 (version 2022.2) for bioinformatic analysis (Bolyen et al., 2019). We used Cutadapt (version 4.3) to demultiplex and remove both adapt and primer sequences from raw paired-end reads (Martin, 2011). The DADA2 (version 1.18.0) plugin (q2-dada2) in QIIME2 pipeline was employed with recommended parameters to successively filter, denoise, merge, and then eliminate singletons and chimeras (Callahan et al., 2016). Reads with low-quality scores (below 20%) and those shorter than 270 bp were filtered to generate Amplicon Sequence Variants (ASVs) feature tables and representative sequences. Each representative sequence was taxonomically classified using a trained Naive Baye Classifier (Quast et al., 2013; Ii et al., 2021). The classifier was trained on 515F/806R amplified fragments using the Sliva 16S rRNA gene database (version 138.1). For downstream analyses, we excluded ASVs identified as eukaryotes, *mitochondria*, and *chloroplast*. To address inconsistent sequencing depth, the ASVs table was rarefied at a minimum threshold based on the lowest read number (21,056) using QIIME2 (q2-feature table plugin). To visualize the community composition of microorganisms with different lifestyles, we selected the taxonomically annotated results in relative abundance > 1 % at both phylum and family taxonomic levels.

### 2.4 Statistical analysis

All data analysis and statistical analysis were conducted and visualized with R Studio software (R Core team, 2024).

#### 2.4.1 Water mass identification

Turbidity is defined as a determinant in locating the sediment front, whose offshore water and inshore water tend to be limpid and turbid, respectively (Ge et al., 2020; Zhou et al., 2020; Du et al., 2025). Salinity is a common physicochemical parameter to identify water clusters (Li et al., 2018; Shen et al., 2018; Li et al., 2023). Therefore, turbidity and salinity are the determinants of water mass identification in the CDF. Moreover, the identification of water mass also necessitates the optimal environmental variables and the number of clustering estimated from CTD profiles. The “fpc” package was a specialized and widely used clustering analysis tool in R (Hennig, 2024). The optimal number and environmental parameters calculated by average silhouette width and Calinski–Harabasz index method can divide the surface, middle and bottom samples to which water mass they belong to (Zhao et al., 2013; Li et al., 2018; Hennig, 2024; Xian et al., 2024). The optimal parameters for surface samples were salinity, turbidity, pH, and temperature, dividing surface samples into four clusters (Figure 1 and Supplementary Table S3). The salinity and turbidity were the optimal parameters dividing the middle samples into two clusters (Figure 1 and Supplementary Table S3). Based on the optimal parameters for bottom waters (salinity, turbidity, and temperature), all samples were divided into three clusters (Figure 1 and Supplementary Table S3).

#### 2.4.2 Environmental correlations with prokaryotic community structure

The relationships between environmental variations and prokaryotic community structures were evaluated through the redundancy analysis (RDA) using the linkET package (Huang, 2021) in R. In this analysis, ASVs data were transformed with the Hellinger method, and physicochemical parameters were selected based on the variation inflation factors (VIFs). The redundant parameters with VIFs exceeding 10 were iteratively removed to minimize the multicollinearity. We performed the mantel test to evaluate the Spearman correlations between prokaryotic community structure and physicochemical variables, with significance tested on 999 Monte Carlo permutation.

#### 2.4.3 Assessing community structure using alpha and beta-diversity indices

Alpha and beta diversity indices were computed at ASV level using QIIME2 (q2-diversity plugin). Alpha diversity indices, insisting of Shannon, phylogenetic diversity (Faith_PD) and Pielou′s evenness index, were computed at ASV level for comparing the biodiversity among three different water masses of PA and FL samples. The statistical significances of differences in both prokaryotes with particular lifestyles and prokaryotes across distinct water masses were detected using the two-sided Wilcoxon signed-rank test and the Mann–Whitney *U*-test based on the properties of pairwise comparison. To evaluate the (dis)similarity of prokaryotic community composition, we performed the non-metric multidimensional scaling (NMDS) analysis (Kruskal, 1964) using the Vegan (version 2.6-8) package in R based on the unweighted-UniFrac distance matrix (Lozupone and Knight, 2005; Oksanen et al., 2024). The statistical differences were detected based on permutational multivariate analysis of variance (PERMANOVA).

### 2.5 Particle-Associated Niche index

To characterize lifestyle preferences, we calculated the Particle-Associated Niche index (PAN index) based on the weighted abundance average of ASVs from PA and FL prokaryotes (Salazar et al., 2015; Mestre et al., 2018; Ma et al., 2024). The PAN index ranged from 0 to 1. When values approaching 0, it indicated a free-living lifestyle, values close to 1 demonstrated a particle-associated lifestyle, and values around 0.5 reflected a transition between PA and FL lifestyles.

### 2.6 Quantification of community assembly mechanisms

We stimulated the neutral community model (NCM) using the MicEco (version 0.9.19) package in R to quantify deterministic and stochastic processes in PA and FL prokaryotic assembly process (Sloan et al., 2006). To partially elucidate the assembly mechanisms in deterministic and stochastic processes, we computed a null model analysis to clarify the specific ecological mechanisms using the iCAMP package (Zhou and Ning, 2017; Ning et al., 2019) in R. The ecological processes included heterogeneous selection (HeS), homogeneous selection (HoS), homogeneous dispersal (HD), dispersal limitation (DL), and drift (DR). The observed ASVs derived from the same ancestor after truncating the phylogenetic tree at a certain phylogenetic distance (as short as necessary) to the root point were grouped into the same group bin. In accordance with the phylogenetic bins, we identified the assembly processes driving the turnovers of each bin (Ning et al., 2020, 2024). The most appropriate phylogenetic tree models for bin clusters were modeled using the optimal algorithms in MEGA (version 11) (Tamura et al., 2021). Eventually, the conducted phylogenetic trees were visualized individually using iTOL (Interactive Tree Of Lifel) for embellishment, annotation and management (Letunic and Bork, 2024).

## 3 Results

### 3.1 Spatial patterns of physicochemical parameters and water mass identification

The frontal effects within the dual fronts system combined with secondary circulation collectively shape the spatial distribution patterns of environmental parameters across the SF, BE, and PF water masses. The drastically decreasing suspended particulate concentration progressively improved light conditions on the offshore side of the sediment front, exhibiting as low turbidity (Supplementary Table S3). From the nearshore SF water mass toward the offshore PF water mass, nutrients (e.g., DIN and DSi) and temperature steadily declined with increasing salinity and pH. A distinct water mass characterized by high salinity, low temperature, and pH developed on the offshore side of the plume front (Supplementary Table S3). Meanwhile, the physicochemical characteristics of the BE water mass exhibit an intermediate between the SF and PF water masses (Supplementary Table S3). Additionally, the relative P-limitation and relative Si-limitation widely existed across three water masses (Table 1). Within the region influenced by CDW, the SF water mass was enriched with sufficient nutrients (DIN: 27.02 ± 12.06 μM, DSi: 28.08 ± 8.55 μM, DIP: 0.28 ± 0.03 μM). Neither relative nor absolute N-limitation was observed except for a large proportion of absolute P-limitation (50%, Table 1) in the SF water mass. Moreover, we found a portion of 14.29% stations experienced the absolute Si-limitation in the BE water masses. The PF water mass displayed relatively high nutrient levels, such as DIN (9.67–11.37 μM) in the surface water at the YEC8 station and DSi (9.99 μM) at the YEC9 station. Nevertheless, the PF water mass still experienced nutritional stress effects (Table 1).

**Table 1:** Proportion of sampling stations with nutrient limitations.

**Nutrient limitation**	**Conditions**	**on-SF/% (8 sites)**	**between-SF-PF/% (7 sites)**	**on-PF/% (18 sites)**
Absolute N-limitation	DIN <1μM	0	0	23.81
Relative N-limitation	DIN/DIP <10 DIN/DSi <1	0	0	42.86
Absolute P-limitation	DIP <0.1μM	50	0	57.146
Relative P-limitation	DIN/DIP > 22 DSi/DIP > 22	62.57	42.86	19.05
Absolute Si-limitation	DSi <2μM	0	14.29	38.10
Relative Si-limitation	DSi/DIN <12 DSi/DIP <10	37.5	42.86	47.62

### 3.2 Alpha and beta diversity in community structure

We obtained a high-quality dataset comprising 1,075,205 sequences from PA and FL water samples. After denoising and quality control, paired-end joining, filtering chimeras, and removing *mitochondria* and *chloroplast*, a total of 2,602 ASVs were generated. The sequencing data achieved a coverage of 99.99 to 100% for prokaryotic species in all samples (Supplementary Table S1), which meant we had effectively captured the prokaryotic community diversity.

**Figure 2:**
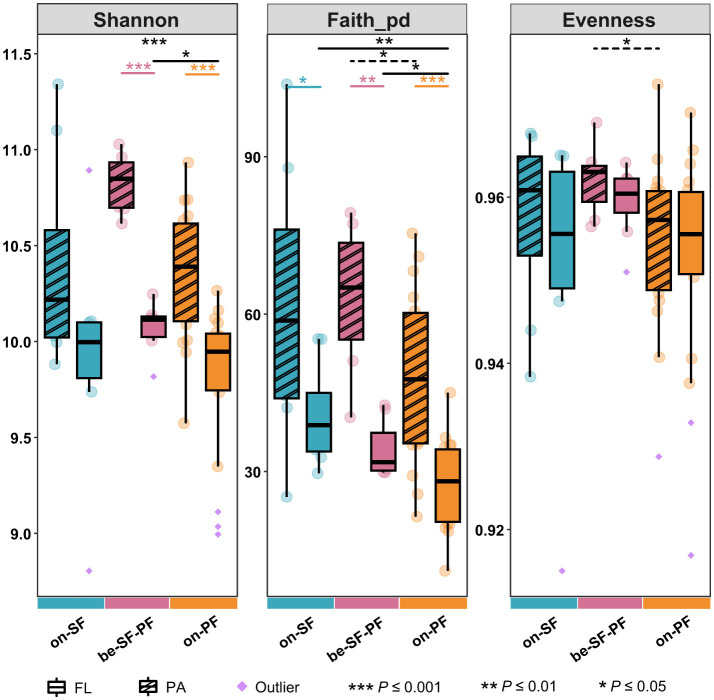
Alpha diversity indices among three water masses in PA and FL communities, such as Shannon index, Faith_pd index and evenness index. Asterisks indicate the significance of difference level based on paired samples *t*-test, where *** represent *P* ≤ 0.001, ** represent *P* ≤ 0.01 and * represents *P* ≤ 0.05.

Across all water masses, the PA prokaryotic community maintained relatively higher richness and evenness than the FL prokaryotic community in the same water mass reflected by Shannon, phylogenetic diversity index (Faith_PD) and Evenness index (Figure 2). The statistical comparisons in the Shannon and Faith_PD index displayed pronounced differences between PA and FL prokaryotic community structures across three water masses (*P* ≤ 0.001), except the Shannon index in the SF water mass (Figure 2). For instance, the PA prokaryotes consistently maintained an elevated Shannon index (9.5–11.35) relative to the FL prokaryotes (8.8–11). The mean Shannon indices for the SF, BE, and PF water masses were 10.166, 10.446, and 10.084, respectively (Supplementary Table S5), indicating a relatively high biodiversity of the BE water mass. Notably, communities in the PF water mass manifested the constrained Shannon index (PA: 9.574–10.033; FL: 8.994–10.265). The Faith_PD further validated statistically distinct differences in communities between the BE and PF water masses (*P* ≤ 0.05, Figure 2). No significant differences in community evenness were detected across three water masses (Figure 2). The PA community in the BE water mass displayed marginally higher evenness compared to that of the PF water mass (*P* ≤ 0.05).

**Figure 3:**
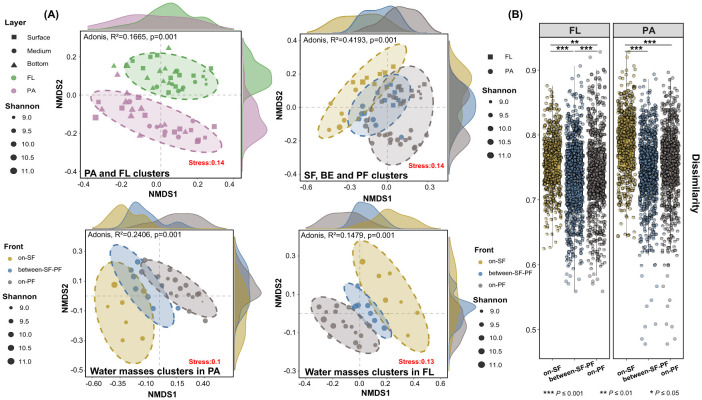
Community structure differences in prokaryotic community structure among three water masses. **(A)** NMDS analysis of prokaryotes with different lifestyle preferences among different water masses; **(B)** Intergroup differences based on the unweighted-UniFrac Distance. Asterisks indicate the significance of difference level based on paired samples *t*-test, where *** represent *P* ≤ 0.001, ** represent *P* ≤ 0.01 and * represents *P* ≤ 0.05.

NMDS analysis was performed to elucidate prokaryotic community structure based on an unweighted-Unifrac distance matrix, stratified by lifestyle preferences and position in the frontal zone (Figure 3A). The results revealed the prokaryotic community exhibited a distinct clustering trend corresponding to lifestyle preferences (confidence <0.9), supported by the ADONIS permutation-based statistical test (*P* ≤ 0.001). In addition, a distinct partitioning was found by the frontal hydrographic activities between PA and FL prokaryotes (Figure 3A). The remarkable dissimilarity in the SF, BE and PF water masses was further confirmed by the Pairwise Samples *t*-test (*P* ≤ 0.001, Figure 3B). Especially, the BE community retained a partial similarity with both SF and PF communities (Figure 3A).

### 3.3 Prokaryotic community composition and lifestyle preferences

**Figure 4:**
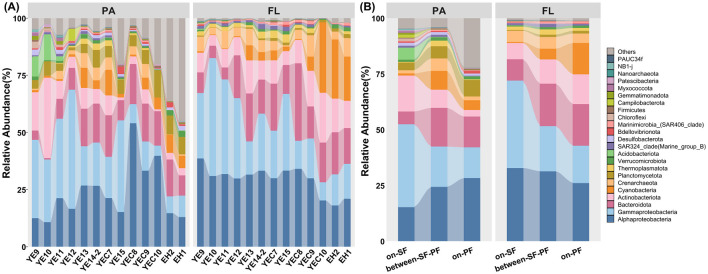
Overview of prokaryotic community compositions in CDF system at phylum level (top 20 phylum in both PA and FL prokaryotic communities). **(A)** The prokaryotic compositions in PA and FL communities across all samples; **(B)** The prokaryotic compositions in PA and FL communities among three water masses. Others represented the prokaryotic phyla except the top 20 phylum.

A total of 69 phyla, 160 classes, 385 orders, 562 families, and 872 genera were identified (Supplementary Table S6). The prokaryotic community in the CDF was predominantly composed of *Proteobacteria* (48.27%), *Bacteroidota* (14.18%), *Actinobacteriota* (8.83%), and *Cyanobacteria* (5.93%, Figure 4A). The most predominant phylum (Figure 4A), *Proteobacteria*, showed high relative abundance in FL and PA communities with 52.56% and 45.49%, respectively (Supplementary Table S6). The class *Alphaproteobacteria* maintained consistent hierarchical dominance across the SF, BE, and PF water masses (Figure 4B). Except for the predominant taxa above, *Acidobacteriota* surpassed *Bacteroidota* in relative abundance in each sample across the water column at stations YE9 and YE10 (Supplementary Figure S2). The *Campilobacterota* exhibits higher relative abundance in the middle sample at YE12 station than in the other samples (Supplementary Figure S2). The prokaryotic community in vertical stratification (e.g., the surface, middle, and bottom water) failed to form a clustering pattern across different stations (Figure 3). Consequently, the significant differences in community combination and distribution patterns were observed in FL and PA samples merged from different depths at each station in the further analysis of community composition (Figure 4).

Further analysis revealed distinct community structures between PA and FL across three water masses (Figure 4B). The PA prokaryotic community included *Planctomycetota* (5.63%), *Acidobacteriota* (1.22%), *Verrucomicrobiota* (1.16%), and *Desulfobacterota* (1.07%). *Thermoplasmatota* (1.63%) was the main phylum of the FL community (Figure 4B). The relative abundance of *Proteobacteria* subsequently declined from the SF to PF water mass in the PA community. At the same time, *Cyanobacteria* exhibited an elevated tendency of relative abundance in the FL community (Figure 4B). Meanwhile, the relative abundance of *Actinobacteriota* decreased with the increasing trend of *Planctomycetota* in the PA community (Figure 4B). The apparent high relative abundance of the prokaryotic phyla except the top 20 phylum indicated there were many kinds of rare prokaryotic taxa in the PA community, particularly in the PF water mass (Figure 4B). In the SF water mass, the sub-dominant group, including *Actinobacteriota, Bacteroidota, Crenarchaeota*, and *Acidobacteriota*, respectively occupied 12.52%, 8.70%, 2.92%, and 2.89% relative abundances, respectively (Supplementary Figure S2). Meanwhile, *Bacteroidota* (15.05%) and *Cyanobacteria* (7.34%) were the sub-dominant groups in the PF water mass (Supplementary Figure S2). Prokaryotic community within the BE water mass showed intermediate compositional characteristics between the SF and PF water masses (Supplementary Figure S2), such as *Bacteroidota* (17.71%), *Actinobacteriota* (9.3%), *Cyanobacteria* (7.06%) and *Crenarchaeota* (5.60%, Supplementary Table S6).

**Figure 5:**
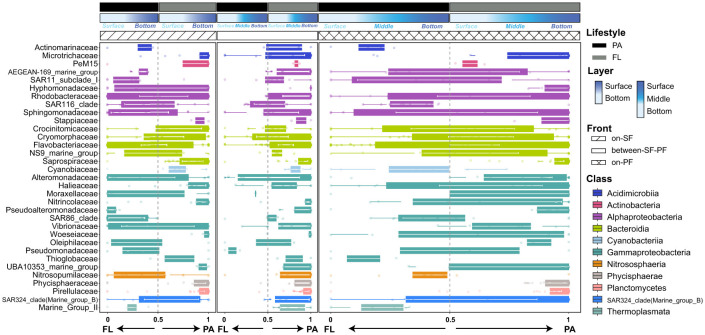
Lifestyles preference in CDF system at family level among three water masses (top 25 family in both PA and FL prokaryotic communities).

To investigate the dynamical adjustment of prokaryotes in response to environmental fluctuations, we combined the community composition with niche preferences defined as the PAN index. More apparent differences in prokaryotes enrichment and contrasting particle preference were shown across distinct water masses at the family level (Figure 5). Most prokaryotes in the BE water mass favored attaching at particles (PAN index >0.5), while those in other water masses showed a transitional tendency between particle-associated and free-living lifestyle (PAN index close to 0.5, Figure 5). *Rhodobacteraceae, Flavobacteriaceae* and *Halieaceae* (15.58%, 8.52%, and 3.73%, Supplementary Table S7) were the dominant prokaryotic taxa distributing across three water masses (Supplementary Figure S3). Corresponding to dynamic environmental fluctuations (e.g., turbidity, salinity, and nutrients), prokaryotic communities experienced the compositional transitions across three water masses (Supplementary Table S3). For instance, only in the PF water mass were *Oleiphilaceae, Hyphomonadaceae, Pseudoalteromonadaceae*, and *Cyanobiaceae* observed to have a relatively high relative abundance (Supplementary Figure S3). *Pseudoalteromonadaceae, Alteromonadaceae, Microtrichaceae*, and *Sphingomonadaceae* uniquely enriched in the FL community of the SF water mass (Supplementary Figure S3). Moreover, in the BE and PF water masses, *Rhodobacteraceae, Flavobacteriaceae, Actinomarinaceae, Alteromonadaceae, Cyanobiaceae* and *Nitrosopumilaceae* consistently switched strategies between particle-associated lifestyle and freely living in the aquatic environments, while displaying stronger preferences for free-living lifestyle in the SF water mass (Figure 5). *Stappiaceae, Pseudomonadales*, and *Saprospiraceae* showed their preferences for particle-associated lifestyle across all water masses (Figure 5). The PAN index of major components in the FL community (Supplementary Figure S3), such as *AEGEAN-169-marine-group, SAR11, SAR116*, and *SAR86*, implied a preference for disassociating from particles (Figure 5). Additionally, *Marine_Group_II* (*MGII*), the dominant archaea, emerged with different lifestyle preferences across three water masses (Figure 5).

### 3.4 Correlations between community structure and physicochemical parameters

**Figure 6:**
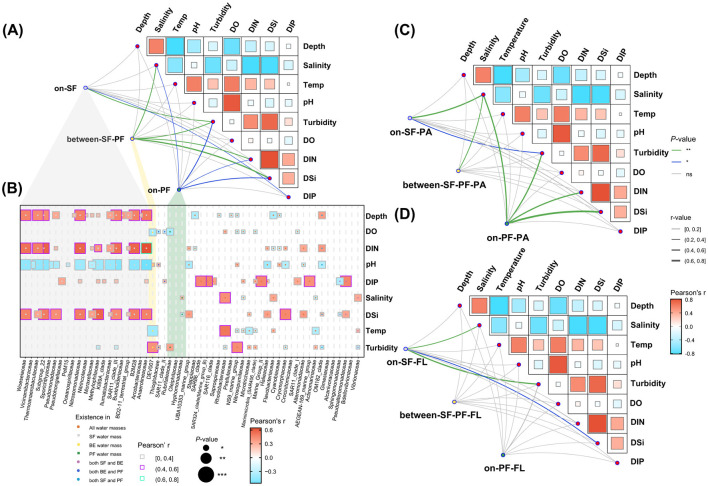
Correlations between community structure and environmental parameters. **(A)** Mantel test of community structure among three water masses; **(B)** The Spearman correlations between the relative abundance of dominant prokaryotes and environmental parameters at family level (the cumulative relative abundance of dominant prokaryotes >90% in each water mass); **(C)** Mantel test of PA community; **(D)** Mantel test of FL community. Asterisks indicate the significance of difference level based on paired samples *t*-test, where *** represent *P* ≤ 0.001, ** represent *P* ≤ 0.01 and * represents *P* ≤ 0.05. Gray shadow indicates prokaryotes only exist in SF, yellow shadow indicates BE, and green shadow indicates PF.

The mantel test results indicated turbidity was the primary influence factor in the prokaryotic community (Figure 6). Salinity, DIN, and DIP collectively played vital roles in shaping the community structure of both the SF and PF water masses (Figure 6A, Supplementary Table S4). Specifically, the prokaryotic community in the SF water mass exhibited prominent relationships with turbidity (*r* ≤ 0.4, *P* ≤ 0.01) and moderate relationships with DIP (*r* ≤ 0.2, *P* ≤ 0.05). DSi emerged as the dominant impact factor of the BE water mass (*r* ≤ 0.6, *P* ≤ 0.01), followed by salinity, turbidity, and DIN (*r* ≤ 0.2, *P* ≤ 0.01). Similar to the BE water mass, salinity, turbidity, DSi, and DIN were key factors impacting the community structure of the PF water mass (Figure 6A). In the SF water mass, the FL community was more responsive to turbidity (*r* ≤ 0.6, *P* ≤ 0.01) and DSi (*r* ≤ 0.4, *P* ≤ 0.05) compared to the PA community (Figures 6C, D). The PA community structures experienced more pronounced influences by environmental parameters in both BE and PF water masses than the FL community (Figures 6C, D). For instance, PA prokaryotes in the BE water mass displayed remarkable associations with salinity (*r* ≤ 0.4, *P* ≤ 0.01), whereas FL prokaryotes lacked substantial associations (Figures 6C, D). Similarly, the PA community in the PF water mass maintained strong associations to environments, in contrast to the FL community which lacked such associations (Figures 6C, D). Additionally, the abundant species in the prokaryotic community of different water masses varied in correlation with environmental parameters (Figure 6B). In the SF water mass, the abundant species showed strong positive correlations with DIN (|*r*| ≤ 0.6, *P* ≤ 0.001), DSi (|*r*| ≤ 0.8, *P* ≤ 0.001), and turbidity (|*r*| ≤ 0.6, *P* ≤ 0.01). Meanwhile, they also had a negative correlation with salinity (|*r*| ≤ 0.4, *P* ≤ 0.01), such as *Anaerolineaceae, SAR11, Nitrosomonadaceae* and *Woeseiaceae* (Figure 6B).

### 3.5 Community assembly processes and ecological driving species

**Figure 7:**
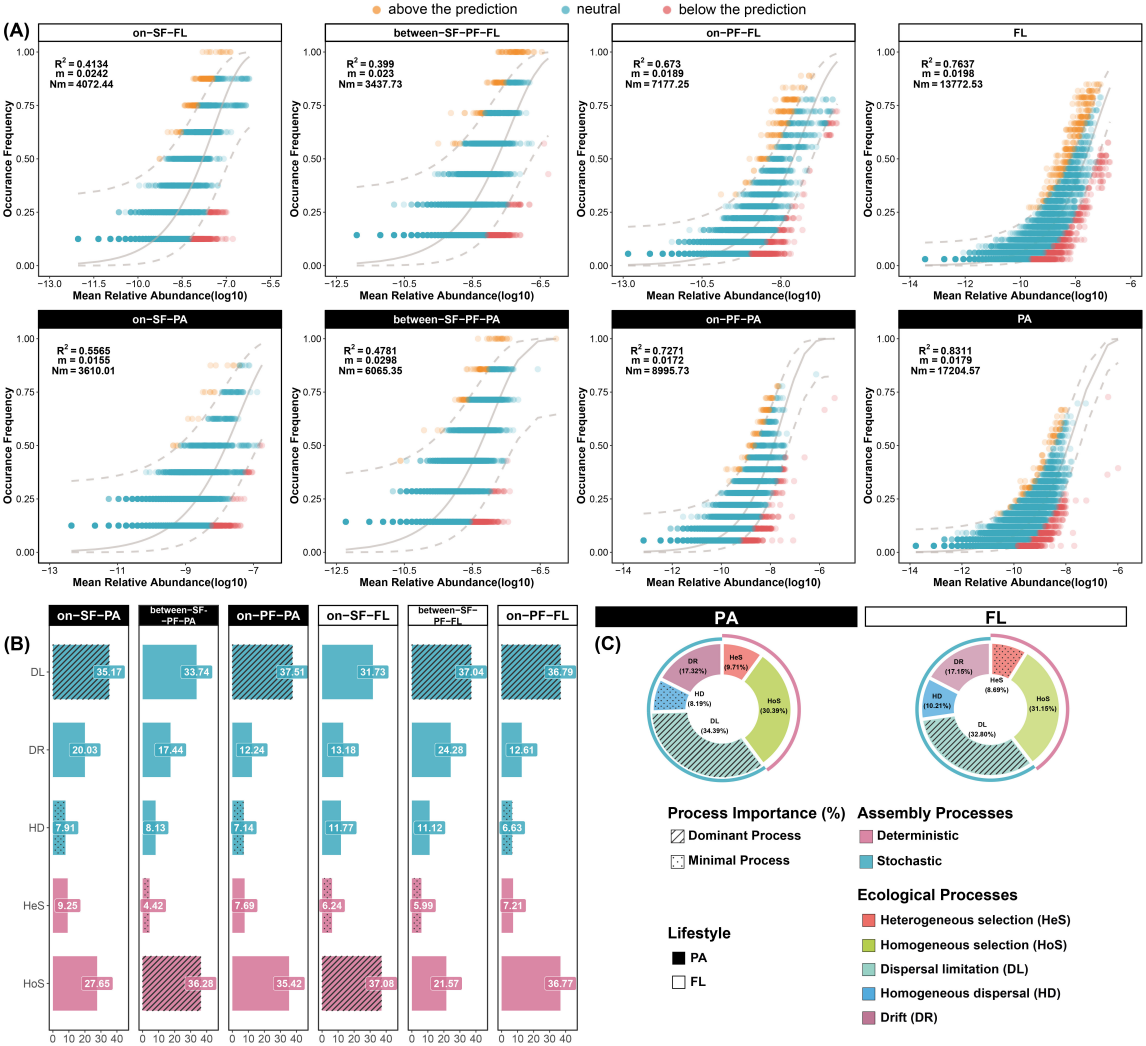
Prokaryotic community assembly processes. **(A)** Stochastic and deterministic processes in FL and PA community assembly processes based on the NCM model. *R*^2^ indicates the fit of the neutral model; Nm represents the estimated community dispersal from the multiplication of N (community size) and m (migration rate). The yellow points represent the observation above the prediction, and the red points represent the observation below the prediction. The light gray solid line denotes the best fit of the neutral model, while the light gray dashed line represents the 95% confidence interval. **(B)** Ecological processes and relative importance among three water masses; **(C)** Ecological processes and relative importance in PA and FL communities. The ecological processes include dispersal limitation (DL), drift (DR), homogeneous dispersal (HD), heterogeneous selection (HeS), and homogeneous selection (HoS).

The neutral community model analysis revealed an excellent fit between the ASV occurrence frequencies and the neutral model in the CDF (*R*^2^ = 0.8861, Supplementary Figure S5). The value of the nice fit also indicated an overall ecological model close to the neutral theoretical pattern (Supplementary Figure S5). Additionally, the PA community showed stronger alignment to neutral ($R_{PA}^{2}$ = 0.8311) compared to FL prokaryotes ($R_{FL}^{2}$ = 0.7637), suggesting the stochastic processes predominantly governed the assembly process of the PA community (Figure 7A). Meanwhile, the consistent patterns emerged across three distinct water masses (Figure 7A). For instance, the PA communities manifested a superior neutral model fit in the SF ($R_{SF-PA}^{2}$ = 0.5565), BE ($R_{BE-PA}^{2}$ = 0.4781), and PF ($R_{PF-PA}^{2}$ = 0.7271) water masses, which exceeded the corresponding fit in the FL communities ($R_{SF-FL}^{2}$ = 0.4134; $R_{BE-FL}^{2}$ = 0.399; $R_{PF-FL}^{2}$ = 0.673). The estimated dispersal extent of the FL community was lower than that of the PA community (*Nm*_*FL*_ = 13,772; *Nm*_*PA*_ = 17,204). Such difference implied the relationship in species migration with diverse lifestyles and enhanced the dispersal capability of the PA prokaryotes (Figure 7A).

The null model analysis quantified the relative contributions of assembly processes. The dispersal limitation (32.80%–34.39%) of stochastic processes and homogeneous selection (30.39%–31.15%) of deterministic processes predominantly controlled the community assembly in the CDF (Figure 7C). The stochastic process was the dominant mechanism in both PA and FL community assemblies (59.9% and 60.16%, Figure 7C), in accordance with the neutral community model (Figure 7A). As the water mass extended further from the CRE, the assembly mechanisms with the most and least contribution varied in different water masses (Figure 7B). As an example, the homogeneous selection functioned as the dominant mechanism for the PA community in the BE water mass and the FL community in the SF water mass. However, the dispersal limitation was the mechanism with the most contribution in other communities. Additionally, the heterogeneous selection represented the least significant ecological process in the FL community assembly (8.69%) (Figure 7C). In contrast, the homogeneous dispersal had the lowest influence on the PA community (8.19%), even less than the heterogeneous selection (9.71%) (Figure 7C). Particularly, the heterogeneous selection and ecological drift exhibited distinctive relative contributions to community assembly across different water masses (Figure 7B). In the PF water mass, the FL community experienced a pronounced effect from heterogeneous selection in comparison with other water masses (Figure 7B). The pronounced contribution of the heterogeneous selection could highlight the broader niche widths of indicator species in the driving mechanism. Meanwhile, ecological drift progressively diminished its contribution to the assembly of the PA community (20.03%–17.44%–12.24%) (Figure 7B). Regarding the FL community, the contribution of ecological drift peaked in the BE water mass (24.28%) (Figure 7B).

**Figure 8:**
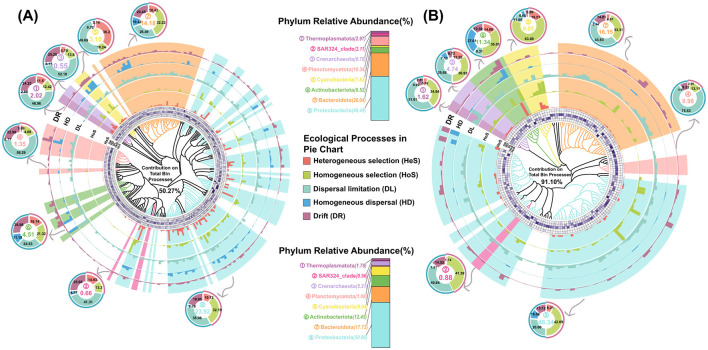
The ecological processes and relative importance of different phylogenetic **(A)** PA-bins and **(B)** FL-bins at family level. The ecological processes include dispersal limitation (DL), drift (DR), homogeneous dispersal (HD), heterogeneous selection (HeS), and homogeneous selection (HoS). The evolutionary tree indicates the phylogenetic relationships of bins. Histograms (five outer annulus) represent ecological processes and absolute contribution to total community assembly. Heatmaps (inner annulus) represent ecological processes and relative importance to each individual bin community assembly. Pie charts represent the ecological processes and relative importance to each phylum community assembly.

Based on the phylogenetic signal thresholds, prokaryotic ASVs from PA and FL communities were classified into 266 and 152 phylogenetic bins, respectively. These bins accounted for 86.97% and 97.91% relative abundance in the PA and FL communities (Figure 8), respectively. By separately cross-referencing the top 25 family from the PA and FL prokaryotic communities, we identified 278 bins belonging to the top 25 family (Figure 8). A total of 162 PA-bins contributed 50.27% to meta-community assembly, predominantly driven by *Proteobacteria* (23.92%) and *Bacteroidota* (14.18%, Figure 8A), and these two dominant participants drove dispersal limitation (35.06%) and homogeneous selection (32.19%, Figure 8A), respectively. *SAR324* (0.66%) and *Crenarchaeota* (0.55%) played crucial roles in ecological drift. In contrast, 116 FL-bins exhibited higher contribution proportion (91.10%, Figure 8B). The coordinated effects from *Cyanobacteria* (83.89%), *Crenarchaeota* (36.93%), *Actinobacteriota* (35.81%) and *Thermoplasmatota* (34.64%) enhanced homogeneous selection (Figure 8B). Specific assembly mechanisms in the sub-community and meta-community were reflected at family level. The PA meta-community was primarily influenced by dispersal limitation, whereas homogeneous selection dominated the FL meta-community assembly (Supplementary Figure S6). Lifestyle transitions between PA and FL dynamically altered the specific mechanism and contribution in the sub-community of assembly drivers. For instance, *Rhodobacteraceae*, a dominant ecological driving species with high contribution in community assembly of both PA and FL lifestyles, showed the reduced contributions in the FL meta-community of the SF water mass (Supplementary Figure S6b). *Sphingomonadaceae* and *Pseudomonadaceae*, freely living in the aquatic environments, drove heterogeneous and homogeneous selection in their sub-community (Supplementary Figure S6b), respectively. While these two contributors transited lifestyle to PA, they shifted synergistically to promote dispersal limitation together with *Microtrichaceae* rather than the deterministic process (Supplementary Figure S6a). Additionally, the assembly of *Moraxellaceae* sub-community in both BE and PF water masses was solely controlled by ecological drift (Supplementary Figure S6a). Intriguingly, *Cyanobacteria* (3.10%) demonstrated divergent assembly patterns owing to lifestyle transitions. FL-*Cyanobacteria* exhibited a weakened contribution in the PF water mass (Figure 8B), compared to the other water mass. Whereas, PA-*Cyanobacteria* activated heterogeneous selection and dispersal limitation (Figure 8A). A similar assembly transition was also observed in the sub-community assembly of *SAR11, NS9*, and *SAR86* clades mediated by three water masses (Supplementary Figure S6b).

## 4 Discussion

### 4.1 Dual fronts function as ecological boundaries in three water masses

The hydrological conditions and lateral variation in environmental gradients in the CDF system cause significant differences in community structure between the SF and PF water masses (Figures 2, 3), and form the unique distribution patterns and lifestyle preferences in the SF, BE, and PF water masses (Figures 4, 5).

The sediment front and plume front restrict the material transport and energy exchange, resulting in the constraints of community structure similarity between the SF and PF water masses (Figure 3A). Simultaneously, the instability of the dual fronts induces the exchanges of water masses across the front, leading to a transitional water mass defined as a critical environmental filter (the BE water mass). While the ecological gradients in the BE water mass enhance the ecosystems' isolation, they still maintain a certain similarity with the other water masses (Figures 3A, B). Such divergences in community structure are particularly evident in the unique combinations of abundant prokaryotes (cumulative relative abundance >90%, Figure 6B). The coexistent prokaryotic taxa in all water masses include *Rhodobacteraceae, Flavobacteriaceae, Cyanobiaceae, Actinomarinaceae* and *Nitrosopumilaceae* (Supplementary Figure S3), consistent with previous studies in the CRE (Ye et al., 2016; Wu et al., 2019; Jiang et al., 2022; Jin et al., 2024). These common marine species play pivotal roles in marine ecosystems, participating in the biogeochemical cycling of carbon, nitrogen, and sulfur (Wu et al., 2013; Simon et al., 2017; Liu et al., 2020). Apart from the coexistent prokaryotes, abundant prokaryotic taxa in the SF water mass show pronounced correlations with turbidity (Figure 6B). This physical parameter from suspended sediment indirectly reflects the correlations with inorganic materials and organic matter, such as chronic nitrogen eutrophication in the SF water mass (Hubeny et al., 2017; Yang et al., 2022; Matos et al., 2024). As an example, *Pseudomonadaceae* possess the capability to degrade terrestrial organic matter (Girard et al., 2023), which can utilize substantial organic matter carried by riverine particles in the SF water mass (Zhou and Ning, 2017; Yang et al., 2022).

The environmental selection and niche competition in distinct microhabitats motivate PA and FL prokaryotes to change their evolutionary trajectories. To minimize competition with other prokaryotes, prokaryotes with different lifestyles can adjust their physiological and morphological traits, genomic composition, physiological function, and survival strategies (Badger et al., 2006; Guider et al., 2024). Compared to the oligotrophic aquatic environments where FL prokaryotes reside, the available suspended particulate matter provides critical substrates for survival and proliferation (Wen et al., 2024; Yu et al., 2025). The dual frontal effects can exacerbate the divergency of PA and FL communities in structure diversity and taxonomic combinations. Previous study have observed the genome of FL-*MGII* contained a large number of genes encoding rhodopsin and opsin, while PA-*MGII* possessed functional genes encoding cell adhesion proteins (Tully, 2019). The FL-*MGII* with photosynthetic strategies could reduce the dependence on particulate organic matter in BE and PF water masses with improved light condition by flocculation (Figure 5). However, the hydrodynamical activities lead to the decoupling of the correlation between FL-*MGII*I and photosynthetic autotrophs, resulting in the maladaptation to FL lifestyle (Figure 5). Therefore, the *MGII* in the SF water mass favoring a particle-associated lifestyle may benefit from its functional genes encoding cell adhesion proteins (Orsi et al., 2015; Wang et al., 2020). The frontal effect can also provide convenient access and functional microhabitats for prokaryotes participating in the biogeochemical cycles, whose metabolic activity often requires an anoxic microenvironment in particulates. For example, *Desulfobacterota*, including *Desulfobulbaceae* and *Desulfuromonadaceae* families, are capable of participating in sulfate reduction and denitrification under anaerobic conditions (Waite et al., 2020; Katie E. Hillyer et al., 2023). Consequently, *Desulfobacterota* usually occurs and accumulates in the sediment-water interface and marine sediments. The relative abundance of *Desulfobacterota* in the SF water masses (1.07%, Figure 4B) benefits from the tidal mixing and resuspension process. The resuspended particles induced by tidal mixing are considered as relative anoxic microenvironments, supporting the colonization and proper progression of biological activities for *Desulfobacterota* (Hallstrøm et al., 2022; Robicheau et al., 2023).

### 4.2 Hydrodynamic activities and lifestyle preferences play essential roles in driving assembly process

Our data demonstrated that stochastic dispersal limitation and deterministic homogeneous selection dominated the assembly process of prokaryotic communities in the CDF (Figure 7, Supplementary Figure S5). Collaboration between intricate turbulent hydrodynamic conditions and lifestyle preferences shifts accounts for the main assembly mechanisms in the CDF system. The current studies have shown the stochastic dispersal rate of the community accelerates with hydrological mixing (Meyerhof et al., 2016; Zhou et al., 2021), which can be applied to the tidal-induced mixing in the SF water mass and the convergency of secondary circulations in the PF water mass. These complicated hydrological activities elevate the contribution of the stochastic process by strengthening the stochastic dispersal rate (Figure 7). The elevated dominance even surpasses the ecological selection imposed by environmental heterogeneity in each water mass, consistent with previous research (Lian et al., 2023; Jin et al., 2024). Consequently, dispersal limitation emerges as the principal mechanism governing prokaryotic community assembly in the dual fronts system (Figure 7).

Hydrological mixing processes in the frontal zone significantly modulate the alterations in water mass (e.g., nutrient gradients and combination and source of organic particulate matter), playing pivotal roles in driving community assembly. In the sediment front, sufficient nutrients (Supplementary Table S3) create favorable niches for prokaryote enrichment, increasing the contribution of homogeneous selection to FL community assembly (Figure 7B). In the plume front, the oligotrophic aquatic environment imposes stringent constraints and selective stress on FL prokaryotic proliferation, which stimulates the compositional divergence in the prokaryotic community. Eventually, intense environmental stress results in a stronger influence on heterogeneous selection (Figure 7B). The elevated contribution of heterogeneous selection also suggests there are indicative species in the PF community, such as *Hyphomonadaceae* with high contributions to community assembly (Supplementary Figure S2). Conversely, organic particulate matter and algal-derived polymers accumulating in the BE water mass mitigate environmental pressures on PA prokaryotes (Li W. et al., 2021; Jin et al., 2024). Therefore, the PF-induced convergence effect can facilitate stable microenvironmental filtering to drive particle-mediated community assembly.

Lifestyle preferences additionally exert profound influences on community assembly in the CDF system, which manifests as the reconfiguration of driving species composition in the sub-community and meta-community. The driving mechanism of lifestyle preferences in assembly mechanisms primarily relates to biodiversity gradients of both meta-community and sub-community. FL prokaryotes, detaching from particles and inhabiting aquatic environments, occupy broader ecological niches and higher spatial turnover rates than PA prokaryotes (Chun et al., 2021; Leu et al., 2022; Zhang et al., 2023). In contrast, the PA prokaryotes are constrained by particle-mediated dispersal within the dual-front system (Figure 7). From a macroscopic perspective, the meta-community theory can account for the different assembly mechanisms of lifestyle preferences (Bjornstad et al., 2009). Such as the declined environmental selection intensity by increasing biomass and community size, eventually modulates the ecological processes in meta-community assembly (Ning et al., 2019; Niu et al., 2022; Lerch et al., 2023). In consequence, the larger meta-community size of the PA prokaryotic community compared to the FL community (Figure 2) alleviates the impacts on the dispersal limitation of the PA community assembly. Such consequences can explain the contrary results based on the neutral community model (Figure 7A). Although the meta-community in the PF water mass experiences a higher limitation in dispersal than the BE water mass, the FL and PA meta-community in the PF water mass disperse wider (Figure 7A). The prokaryotic community in the PF water mass possesses a more extensive meta-community than the other water mass, which spans vertically across the plume front (Figure 1). In consequence, the larger meta-community size in the PF water mass alleviates the impacts on dispersal limitation. Eventually, the counteracting effect explains the significant influence of dispersal limitation, but low dispersal extent of the meta-community mentioned above (Figure 7A). From a microscopic perspective, the relative abundance of specific prokaryotic taxa is the main reason to drive the assembly processes of the sub-community. For instance, the dominance in relative abundance and exceptional taxonomic diversity (Figures 8A, B) directly make *Proteobacteria* a major driver of community assembly in the CDF (Vellend et al., 2014; Evans et al., 2017; Xun et al., 2019). Including *Alphaproteobacteria* and *Gammaproteobacteria*, these families play pivotal ecological roles in community assembly when associated with particulates or freely living in the aquatic environment (Supplementary Figure S3). Moreover, the community with high abundance is easily prone to induce disproportionate richness, resulting in ecological drift and dispersal limitation collectively driving the assembly of *Proteobacteria* sub-community (e.g., halotolerant *Rhodobacteraceae*, Figure 8).

The intensive hydrodynamic activities stimulate the attachment-detachment on particulates of prokaryotes by providing mechanical energy and constraining the availability of substrate. Eventually, this synergistic mechanism between hydrodynamic activities and lifestyle preferences exerts a vital influence on the community assembly processes. For instance, localized accumulation in both SF and BE water masses causes the disproportionate distribution of particulates across the water column (Supplementary Table S3), causing pronounced disparities in abundance (Evans et al., 2017; Xun et al., 2019). Eventually, such structural imbalances produce a high contribution in ecological drift progressively from the SF water mass to the PF water mass (Figure 8B). The increasing contributions in ecological drift suggest species extinction, immigration, and recolonization events substantially influence the community assembly processes (Ning et al., 2020). FL prokaryotes exhibit heightened environmental sensitivity, their detachment from particulates intensifies homogeneous dispersal and homogeneous selection in community assembly (Banerjee et al., 2016). Conversely, biofilm-forming PA prokaryotes are buffered from environmental fluctuations, reducing the importance of stochastic dispersal and increasing their susceptibility to dispersal limitation (Banerjee et al., 2016), such as *Sphingomonadaceae* (Supplementary Figures S6a, b). In the SF water mass, a rapid increase in suspended particulate matter reduces light penetration and restricts the survival conditions of *Sphingomonadaceae* (Ivanova et al., 2020; Zhou et al., 2020; Li W. et al., 2021). In consequence, *Sphingomonadaceae* taxa are unable to thrive in the FL community (Figure 5), and merely attach to particulates to convert organic substrates into essential biomolecules. The metabolic function traits of *Sphingomonadaceae* increase its contribution to dispersal limitation in PA community assembly (Supplementary Figure S6). However, the tidal-reduced resuspension processes in the SF water mass drive prokaryotes to shift lifestyle to freely floating, eventually diminishing the importance of dispersal limitation (Ohore et al., 2022). In the SF water mass, the particle-associated lifestyle of *Cyanobacteria* restricts cell mobility and reduces the susceptibility to environmental heterogeneity. In consequence, the tendency of particle-associated lifestyle weakens the ability of PA-*Cyanobacteria* to adjust to selection pressures (Figure 8B) and elevates the relative importance of dispersal limitation and heterogeneous selection (Supplementary Figure S6).

## 5 Conclusion

Our study reveals the unique compositional characteristics of prokaryotic communities in water masses on either side of the Yangtze River Estuary Dual Fronts system and highlights the role of hydrodynamical activities and lifestyle preferences in driving the sub-community and meta-community assembly. The environmental gradients caused by the lateral variations and secondary circulations result in significant differences in the prokaryotic community of the dual fronts system. Additionally, the complicated hydrodynamical activities reorganize the combinations in the particle-associated (PA) and free-living (FL) prokaryotic communities. More importantly, we meticulously emphasize the double mechanism of stochastic dispersal limitation and deterministic homogeneous selection of dominating prokaryotic community assembly. The hydrological mixing processes together with lifestyle preferences collaboratively drive different assembly mechanisms for the meta-communities and sub-communities. FL prokaryotes with high environmental sensitivity promote homogeneous dispersal and selection in community assembly. Conversely, the biofilm-forming PA prokaryotes are buffered against environmental fluctuations, reducing the influence of stochastic dispersal. The intensive hydrodynamic activities stimulate the rapid attachment-detachment dynamics of prokaryotes on particulates, altering the assembly mechanisms and driving species distribution (e.g., FL and PA-*Cyanobacteria*). In the SF water mass, the sufficient nutrients and substrates create favorable niches for prokaryotic enrichment, driving homogeneous selection in the FL community assembly. However, the tidal-induced resuspension processes shift PA prokaryotes to a freely living lifestyle in the SF water mass, diminishing the importance of dispersal limitation, such as the sub-community of *Microtrichaceae*. In the BE water mass, the accumulation of organic particulate matter and algal-derived polymers imposed by the PF-induced convergence effect can mitigate the environmental pressures on PA prokaryotes, driving environmental filtering in PA community assembly. In the PF water mass, the oligotrophic aquatic environment imposes stringent constraints and selective stress on FL prokaryotic proliferation, driving heterogeneous selection in FL community assembly, which additionally suggests the presence of indicative species (e.g., *Hyphomonadaceae*). Such biological effects in microbial responses further verify the natural barrier function of the sediment front (SF) and plume front (PF) in demarcating biogeographic boundaries.

## Data Availability

The original contributions presented in the study are publicly available. This data can be found here: https://www.ncbi.nlm.nih.gov, accession number PRJNA1249246.
