# Supplementary material for: Hydrodynamic activities and lifestyle preferences synergistically drive prokaryotic community assembly processes in the dual fronts system of the Yangtze River Estuary
Source: Front Microbiol. 2025 Jul 31;16:1610617. doi: 10.3389/fmicb.2025.1610617 (PMC12350477; doi:10.3389/fmicb.2025.1610617)
Supplement: Supplementary file 1 [file Supplementary_file_1.docx]

Supplementary Material

# 1 Supplementary Data

The datasets presented for this study can be found in online repositories. The names of the repository/repositories and accession number (s) can be found at: <https://www.ncbi.nlm.nih.gov/bioproject/PRJNA1249246>.

# 2 Supplementary Figures and Tables

## 2.1 Supplementary Figures

**Supplementary Figure S1.** Planar spatial distribution patterns of physicochemical parameters (e.g., temperature (℃), salinity (PSU), pH, turbidity (FNU), DSi (μM), DIP (μM), DIN (μM)) in the surface water layer of CDF system. DO means the dissolved oxygen, DIN means the dissolved inorganic nitrogen (NO3− + NO2− + NH4+), DSi means the dissolved silicate, and DIP means the dissolved inorganic phosphate.

**Supplementary Figure S2.** Overview of prokaryotic community compositions across different water masses at phylum level (top 20 phyla in both PA and FL prokaryotic communities). Others represented the prokaryotic phyla except the top 20 phylum.

Supplementary Figure S3. Prokaryotic community compositions in the CDF system at family level among three water masses (top 25 families in both PA and FL prokaryotic communities).

**Supplementary Figure S4.** RDA analysis of the prokaryotic community structure and the physiochemical parameters selected by VIFs. The arrows indicate the direction and magnitude of the measurable variables associated with prokaryotic community structures. The colors of the arrows indicate the correlation of the permutation test between the prokaryotic community and the environmental parameters, the purple arrow indicates *P* ≤ 0.001: ***, the green arrow indicates *P* ≤ 0.01: **, the blue arrow indicates *P* ≤ 0.05: *, and the grey arrow indicates *P* > 0.05: ns.

**Supplementary Figure S5.** Stochastic and deterministic processes in the overall community assembly processes based on the NCM model. R² indicates the goodness fit of the neutral model; Nm represents the estimated community dispersal from the multiplication of N (community size) and m (migration rate). The yellow points represent the observation above the prediction, and the red points represent the observation below the prediction. The light gray solid line denotes the best fit of the neutral model, while the light gray dashed line represents the 95% confidence interval.

**Supplementary Figure S6a.** The ecological processes and relative importance of different phylogenetic PA-bins at family level (top 20 family in both PA and FL prokaryotic communities), including dispersal limitation (DL), drift (DR), homogeneous dispersal (HD), heterogeneous selection (HeS), and homogeneous selection (HoS).

**Supplementary Figure S6b.** The ecological processes and relative importance of different phylogenetic FL-bins at family level (top 20 family in FL prokaryotic communities), including dispersal limitation (DL), drift (DR), homogeneous dispersal (HD), heterogeneous selection (HeS) and, homogeneous selection (HoS).

**Supplementary Figure S6.** The ecological processes and relative importance of different phylogenetic (A) PA-bins and (B) FL-bins at family level (top 20 family in both PA and FL prokaryotic communities). The ecological processes include dispersal limitation (DL), drift (DR), homogeneous dispersal (HD), heterogeneous selection (HeS) and, homogeneous selection (HoS). Histograms represent ecological processes and absolute contribution to total community assembly. Heatmaps represent ecological processes and relative importance to each individual bin community assembly. Pie charts represent the ecological processes and relative importance of each phylum community assembly.

## 2.2 Supplementary Tables

**Supplementary Table S1.** Numeric data summarized by raw sequence, denoised sequence, and ASV frequency of each sample.

| **Sample-ID** | **Raw Sequence** | **Filtered** | **Denoised** | **Merged** | **Non-chimeric** | **ASVs** |
| --- | --- | --- | --- | --- | --- | --- |
| YE9-1-0.22 μm | 44445 | 27375 | 24925 | 21127 | 21051 | 277 |
| YE9-2-0.22 μm | 55152 | 33736 | 29755 | 23615 | 23615 | 359 |
| YE10-1-0.22 μm | 53594 | 33847 | 31407 | 27820 | 27814 | 247 |
| YE10-2-0.22 μm | 53358 | 34482 | 33360 | 31160 | 31024 | 148 |
| YE11-1-0.22 μm | 54215 | 33558 | 31457 | 27654 | 27618 | 202 |
| YE11-2-0.22 μm | 94016 | 59105 | 54823 | 48179 | 47968 | 343 |
| YE12-1-0.22 μm | 60999 | 38044 | 35008 | 28686 | 28636 | 189 |
| YE12-2-0.22 μm | 58011 | 36898 | 33919 | 28601 | 28468 | 203 |
| YE13-1-0.22 μm | 63112 | 40603 | 38751 | 34840 | 34768 | 191 |
| YE13-2-0.22 μm | 53116 | 31751 | 29737 | 25088 | 25083 | 171 |
| YE14-2-1-0.22 μm | 58889 | 37020 | 34863 | 30847 | 30781 | 196 |
| YE14-2-2-0.22 μm | 56418 | 35604 | 33769 | 29983 | 29882 | 196 |
| YEC7-1-0.22 μm | 55550 | 34486 | 32667 | 29041 | 28926 | 190 |
| YEC7-2-0.22 μm | 68025 | 42890 | 40496 | 35725 | 35589 | 245 |
| YEC7-3-0.22 μm | 59915 | 37885 | 35112 | 29964 | 29926 | 272 |
| YE15-1-0.22 μm | 66261 | 40892 | 38154 | 32557 | 32385 | 187 |
| YE15-2-0.22 μm | 65482 | 40878 | 38348 | 32679 | 32603 | 192 |
| YE15-3-0.22 μm | 66041 | 41593 | 38680 | 33844 | 33824 | 205 |
| EH1-1-0.22 μm | 53209 | 32851 | 31860 | 29233 | 29198 | 114 |
| EH1-2-0.22 μm | 60930 | 38571 | 36398 | 32750 | 32665 | 211 |
| EH1-3-0.22 μm | 60060 | 36869 | 34607 | 29768 | 29755 | 221 |
| EH2-1-0.22 μm | 51994 | 31246 | 30227 | 27617 | 27447 | 100 |
| EH2-2-0.22 μm | 60239 | 36992 | 34937 | 30674 | 30620 | 178 |
| EH2-3-0.22 μm | 50720 | 31740 | 30312 | 27033 | 27007 | 177 |
| YEC9-1-0.22 μm | 52107 | 32459 | 30371 | 26317 | 26268 | 78 |
| YEC9-2-0.22 μm | 59242 | 35786 | 33220 | 28090 | 28074 | 127 |
| YEC9-3-0.22 μm | 50319 | 31864 | 29571 | 24854 | 24804 | 226 |
| YEC8-1-0.22 μm | 43065 | 27246 | 26270 | 24398 | 24246 | 150 |
| YEC8-2-0.22 μm | 45026 | 27651 | 26693 | 23178 | 23037 | 220 |
| YEC8-3-0.22 μm | 49407 | 30128 | 28263 | 23994 | 23991 | 283 |
| YEC10-1-0.22 μm | 52536 | 32478 | 30693 | 26403 | 26138 | 110 |
| YEC10-2-0.22 μm | 47940 | 29124 | 27624 | 23728 | 23713 | 134 |
| YEC10-3-0.22 μm | 48497 | 29736 | 28282 | 24020 | 23980 | 128 |
| YE9-S-3 μm | 47943 | 39194 | 37124 | 29361 | 29093 | 435 |
| YE9-D-3 μm | 65266 | 53113 | 46896 | 32910 | 32860 | 669 |
| YE10-S-3 μm | 69398 | 52730 | 51952 | 49687 | 49335 | 157 |
| YE10-D-3 μm | 45631 | 37838 | 36382 | 31474 | 31396 | 318 |
| YE11-S-3 μm | 133620 | 99740 | 90640 | 76054 | 75239 | 761 |
| YE11-D-3 μm | 70649 | 50453 | 46599 | 36873 | 36659 | 549 |
| YE12-S-3 μm | 83803 | 71060 | 66797 | 60100 | 59759 | 360 |
| YE12-D-3 μm | 66645 | 57988 | 54738 | 42465 | 42018 | 270 |
| YE13-S-3 μm | 86393 | 73003 | 66899 | 56213 | 56003 | 548 |
| YE13-D-3 μm | 84654 | 69842 | 64690 | 55254 | 54846 | 408 |
| YE14-2-S-3 μm | 51172 | 43969 | 41871 | 38692 | 38545 | 228 |
| YE14-2-D-3 μm | 81533 | 68779 | 63610 | 54902 | 54733 | 415 |
| YEC7-S-3 μm | 69567 | 59362 | 56374 | 50370 | 50139 | 344 |
| YEC7-M-3 μm | 77226 | 65386 | 60180 | 51437 | 51258 | 480 |
| YEC7-D-3 μm | 74722 | 62854 | 57986 | 50406 | 50258 | 537 |
| YE15-S-3 μm | 55617 | 47117 | 44174 | 39294 | 39203 | 269 |
| YE15-M-3 μm | 60902 | 50969 | 47895 | 41191 | 41034 | 370 |
| YE15-D-3 μm | 78734 | 68171 | 65149 | 58116 | 57880 | 353 |
| EH1-S-3 μm | 51336 | 43412 | 42558 | 40386 | 40155 | 137 |
| EH1-M-3 μm | 48087 | 39780 | 38068 | 34119 | 34069 | 303 |
| EH1-D-3 μm | 61462 | 50700 | 47181 | 39844 | 39825 | 441 |
| EH2-S-3 μm | 47832 | 39813 | 38516 | 35341 | 35182 | 160 |
| EH2-M-3 μm | 70711 | 59573 | 55706 | 48229 | 47917 | 374 |
| EH2-D-3 μm | 47372 | 39803 | 38232 | 34381 | 34286 | 299 |
| YEC8-S-3 μm | 59296 | 50263 | 49277 | 46890 | 46381 | 109 |
| YEC8-M-3 μm | 45499 | 38955 | 38064 | 36072 | 35941 | 140 |
| YEC8-D-3 μm | 81805 | 68233 | 62891 | 52543 | 52407 | 539 |
| YEC9-S-3 μm | 55850 | 47884 | 46411 | 42770 | 42673 | 187 |
| YEC9-M-3 μm | 57899 | 48197 | 44975 | 37721 | 37566 | 411 |
| YEC9-D-3 μm | 80891 | 69322 | 64024 | 55645 | 55406 | 494 |
| YEC10-S-3 μm | 57637 | 48273 | 46549 | 43338 | 43042 | 178 |
| YEC10-M-3 μm | 54388 | 46284 | 43824 | 39148 | 38934 | 252 |
| YEC10-D-3 μm | 47236 | 40013 | 38335 | 35058 | 34872 | 187 |

*^a^* "1" & "S" of the Sample-ID represent surface samples, and "2" & "M" represent middle samples, "3" & "D" represent bottom samples.

*^b^* "0.22 μm" represents the free-living samples, and “3 μm” represents the particle-associated samples.

**Supplementary Table S2.** Overview of samples in each station at surface, middle, and bottom depth.

| **Front** | **Station** | **Sample-ID** | **Longitude** | **Latitude** | **Depth** |
| --- | --- | --- | --- | --- | --- |
|  |  |  | **(°E)** | **(°N)** | **(m)** |
| on-SF | YE9 | YE9-1 | 122.1494 | 31.01894 | 2 |
|  |  | YE9-2 |  |  | 6 |
|  | YE10 | YE10-1 | 122.2293 | 30.9572 | 2 |
|  |  | YE10-2 |  |  | 6 |
|  | YE11 | YE11-1 | 122.4004 | 30.9085 | 2 |
|  |  | YE11-2 |  |  | 9 |
|  | YE12 | YE12-1 | 122.4732 | 30.9000 | 2 |
|  |  | YE12-2 |  |  | 11 |
| between-SF-PF | YE13 | YE13-1 | 122.6393 | 30.8619 | 2 |
|  |  | YE13-2 |  |  | 23 |
|  | YE14-2 | YE14-2-1 | 122.8535 | 30.7983 | 2 |
|  |  | YE14-2-2 |  |  | 23 |
|  | YEC7 | YEC7-1 | 122.7096 | 30.2245 | 2 |
|  |  | YEC7-2 |  |  | 12 |
|  |  | YEC7-3 |  |  | 25 |
| on-PF | YE15 | YE15-1 | 122.9372 | 30.7129 | 2 |
|  |  | YE15-2 |  |  | 20 |
|  |  | YE15-3 |  |  | 40 |
|  | EH1 | EH1-1 | 123.0838 | 30.5075 | 3 |
|  |  | EH1-2 |  |  | 35 |
|  |  | EH1-3 |  |  | 55 |
|  | EH2 | EH2-1 | 122.9358 | 30.3198 | 2 |
|  |  | EH2-2 |  |  | 30 |
|  |  | EH2-3 |  |  | 47 |
|  | YEC8 | YEC8-1 | 122.7433 | 30.0232 | 2 |
|  |  | YEC8-2 |  |  | 22 |
|  |  | YEC8-3 |  |  | 33 |
|  | YEC9 | YEC9-1 | 122.7236 | 29.7588 | 2 |
|  |  | YEC9-2 |  |  | 24 |
|  |  | YEC9-3 |  |  | 39 |
|  | YEC10 | YEC10-1 | 122.5866 | 29.6182 | 2 |
|  |  | YEC10-2 |  |  | 20 |
|  |  | YEC10-3 |  |  | 35 |

*^a^* "on-SF" represents the station in the water mass of the sediment front, "between-SF-PF" represents the station in the water mass between the sediment front and the plume front, and "on-PF" represents the station in the water mass of the plume front.

*^b^* Salinity, turbidity, pH, and temperature are the optimal parameters for clustering the surface samples; salinity and turbidity are the optimal parameters for clustering the middle samples; salinity, turbidity, and temperature are the optimal parameters for clustering the bottom samples.

**Supplementary Table S3.** Overview of physicochemical parameters in all samples and three water masses.

| **Sample-ID** | **Salinity**  **(PSU)** | **Temp**  **(℃)** | **pH** | **Turbidity**  **(FNU)** | **DO**  **(mg/l)** | **DSi**  **(μM)** | **DIN^-^**  **(μM)** | **DIP^-^**  **(μM)** |
| --- | --- | --- | --- | --- | --- | --- | --- | --- |
| on-SF | 22.33 ± 2.81 | 28 ± 0.51 | 7.9 ± 0.06 | 167.77 ± 112.18 | 5.09 ± 0.38 | 27.02 ± 12.06 | 28.08 ± 8.55 | 0.28 ± 0.03 |
| between-SF-PF | 32.23 ± 1.63 | 25.19 ± 2.34 | 7.96 ± 0.16 | 23.45 ± 32.78 | 4.89 ± 1.34 | 6.24 ± 4.05 | 15.51 ± 5.61 | 0.32 ± 0.1 |
| on-PF | 35.07 ± 1.65 | 23.83 ± 4.03 | 7.92 ± 0.16 | 7.34 ± 11.06 | 4.66 ± 2.15 | 6.92 ± 5.07 | 4.64 ± 3.99 | 0.16 ± 0.08 |
| YE9-1 | 19.29 | 28.314 | 7.85 | 183.82 | 5.11 | 35.6 | 26.39 | 0.33 |
| YE9-2 | 19.27 | 28.332 | 7.85 | 279.58 | 4.88 | 49.71 | 31.23 | 0.29 |
| YE10-1 | 21.12 | 28.217 | 7.86 | 48.23 | 4.83 | 14.84 | 16.86 | N.D. |
| YE10-2 | 21.08 | 28.216 | 7.88 | 269.56 | 4.96 | 31.54 | 25.18 | 0.26 |
| YE11-1 | 21.19 | 27.987 | 7.89 | 34.77 | 5.32 | 34.97 | 43.9 | 0.3 |
| YE11-2 | 23.34 | 28.261 | 7.89 | 285.39 | 4.93 | 17.02 | 28.25 | N.D. |
| YE12-1 | 25.95 | 27.931 | 8.04 | 3.69 | 5.98 | 18.32 | 35.96 | 0.24 |
| YE12-2 | 27.42 | 26.7025 | 7.95 | 237.14 | 4.685 | 14.12 | 16.88 | N.D. |
| YE13-1 | N.D. | N.D. | N.D. | N.D. | N.D. | 10.88 | 16 | 0.18 |
| YE13-2 | N.D. | N.D. | N.D. | N.D. | N.D. | 7.88 | 15.64 | N.D. |
| YE14-2-1 | 30.37 | 27.631 | 8.26 | 0.17 | 6.91 | 1.46 | 6.09 | 0.38 |
| YE14-2-2 | 34.81 | 21.102 | 7.78 | 51.64 | 2.85 | 2.32 | 7.52 | 0.42 |
| YEC7-1 | 30.81 | 26.747 | 7.94 | 1.91 | 5.25 | 3.65 | 22.14 | 0.24 |
| YEC7-2 | 32.05 | 25.933 | 7.93 | 3.27 | 5.08 | 8.65 | 19.2 | 0.22 |
| YEC7-3 | 33.3 | 23.81 | 7.92 | 80.44 | 4.15 | 12.73 | 18.91 | 0.42 |
| YE15-1 | 32.35 | 28.636 | 8.06 | 0.67 | 6.04 | N.D. | 2.37 | N.D. |
| YE15-2 | 36.01 | 24.152 | 7.92 | 0.675 | 4.755 | 3.24 | 4.11 | 0.32 |
| YE15-3 | 36.21 | 19.182 | 7.84 | 10.16 | 3.5 | N.D. | 1.28 | N.D. |
| EH1-1 | 36.2575 | 18.7925 | 7.775 | 0.925 | 6.03 | N.D. | 0.76 | N.D. |
| EH1-2 | 36.11 | 20.453 | 7.82 | 0.66 | 4.56 | 8.36 | 7.4 | N.D. |
| EH1-3 | 36.28 | 19.7645 | 8.05 | 3.57 | 3 | 11.61 | 9.93 | N.D. |
| EH2-1 | 34.78 | 29.5195 | 8.095 | 0.74 | 6.315 | N.D. | 0.64 | N.D. |
| EH2-2 | 36.214 | 19.4558 | 7.738 | 2.92 | 2.192 | 6.24 | 4.81 | 0.24 |
| EH2-3 | 36.12 | 19.251 | 7.74 | 22.31 | 2.18 | 8.11 | 10.49 | 0.17 |
| YEC8-1 | 29.83 | 28.16 | 8.29 | 0.52 | 10.91 | 0.57 | 9.67 | N.D. |
| YEC8-2 | 35.81 | 25.81 | 7.79 | 4.28 | 3.25 | 2.11 | 9.81 | N.D. |
| YEC8-3 | 35.62 | 21.051 | 7.76 | 33.65 | 2.62 | 19.05 | 11.37 | 0.19 |
| YEC9-1 | 34.17 | 29.026 | 8.1 | 0.84 | 6.5 | 9.99 | 0.51 | 0.02 |
| YEC9-2 | 35.89 | 26.889 | 7.95 | 0.591667 | 5.018 | 2.32 | 2.89 | 0.24 |
| YEC9-3 | 35.785 | 20.0575 | 7.77 | 35.785 | 2.875 | 5.4 | 5.78 | 0.21 |
| YEC10-1 | 33.56 | 29.398 | 8.12 | 0.8 | 6.94 | N.D. | 0.35 | N.D. |
| YEC10-2 | 34.64 | 26.81 | 7.94 | 2.68 | 2.79 | N.D. | 1.16 | N.D. |
| YEC10-3 | 35.66 | 22.569 | 7.77 | 10.38 | 4.44 | N.D. | 0.27 | N.D. |

*^a^* N.D. represents not done.

*^b^* "1" of the Sample-ID represents surface samples, and "2" represents middle samples, "3" represents bottom samples. "on-SF" represents the station in the water mass of the sediment front, "between-SF-PF" represents the station in the water mass between the sediment front and the plume front, and "on-PF" represents the station in the water mass of the plume front.

*^c^* DO represents the dissolved oxygen, DIN represents the dissolved inorganic nitrogen (NO_3_^−^ + NO_2_^−^ + NH_4_^+^), DSi represents the dissolved silicate, and DIP represents the dissolved inorganic phosphate.

**Supplementary Table S4.** The Spearman correlation test between the prokaryotic community structures and physicochemical parameters among different water masses.

| **Group** | **Physicochemical**  **factors** | **r** | ***p.value*** | **rd** | **pd** |
| --- | --- | --- | --- | --- | --- |
| on-SF-FL | Depth | -0.08956 | 0.866 | ≤0.2 | >0.1 |
|  | Shannon | 0.23402 | 0.102 | >0.2 | >0.1 |
|  | Salinity | 0.373336 | 0.007 | >0.2 | <0.01 |
|  | Temperature | -0.07816 | 0.792 | ≤0.2 | >0.1 |
|  | pH | -0.15161 | 0.976 | ≤0.2 | >0.1 |
|  | Turbidity | 0.574536 | 0.003 | >0.4 | <0.01 |
|  | DO | -0.16019 | 0.982 | ≤0.2 | >0.1 |
|  | DIN | 0.233809 | 0.049 | >0.2 | <0.05 |
|  | DSI | 0.339208 | 0.049 | >0.2 | <0.05 |
|  | DIP | -0.02707 | 0.5 | ≤0.2 | >0.1 |
| between-SF-PF-FL | Depth | 0.172541 | 0.084 | ≤0.2 | >0.1 |
|  | Shannon | -0.12746 | 0.808 | ≤0.2 | >0.1 |
|  | Salinity | 0.000516 | 0.424 | ≤0.2 | >0.1 |
|  | Temperature | 0.133149 | 0.064 | ≤0.2 | >0.1 |
|  | pH | -0.14125 | 0.95 | ≤0.2 | >0.1 |
|  | Turbidity | 0.007759 | 0.339 | ≤0.2 | >0.1 |
|  | DO | -0.08149 | 0.709 | ≤0.2 | >0.1 |
|  | DIN | -0.0358 | 0.485 | ≤0.2 | >0.1 |
|  | DSI | 0.032845 | 0.298 | ≤0.2 | >0.1 |
|  | DIP | 0.029497 | 0.272 | ≤0.2 | >0.1 |
| on-PF-FL | Depth | -0.07349 | 0.837 | ≤0.2 | >0.1 |
|  | Shannon | 0.007869 | 0.306 | ≤0.2 | >0.1 |
|  | Salinity | 0.004906 | 0.325 | ≤0.2 | >0.1 |
|  | Temperature | 0.006693 | 0.389 | ≤0.2 | >0.1 |
|  | pH | -0.07954 | 0.692 | ≤0.2 | >0.1 |
|  | Turbidity | 0.237815 | 0.116 | >0.2 | >0.1 |
|  | DO | -0.09788 | 0.788 | ≤0.2 | >0.1 |
|  | DIN | -0.06136 | 0.652 | ≤0.2 | >0.1 |
|  | DSI | -0.06382 | 0.533 | ≤0.2 | >0.1 |
|  | DIP | -0.00924 | 0.348 | ≤0.2 | >0.1 |
| on-SF-PA | Depth | -0.09295 | 0.893 | ≤0.2 | >0.05 |
|  | Shannon | 0.021211 | 0.345 | ≤0.2 | >0.05 |
|  | Salinity | 0.433387 | 0.001 | >0.4 | <0.01 |
|  | Temperature | -0.09522 | 0.899 | ≤0.2 | >0.05 |
|  | pH | -0.16699 | 0.994 | ≤0.2 | >0.05 |
|  | Turbidity | 0.335346 | 0.039 | >0.2 | <0.05 |
|  | DO | -0.18086 | 1 | ≤0.2 | >0.05 |
|  | DIN | 0.168004 | 0.108 | ≤0.2 | >0.05 |
|  | DSI | 0.206845 | 0.105 | >0.2 | >0.05 |
|  | DIP | -0.00916 | 0.417 | ≤0.2 | >0.05 |
| between-SF-PF-PA | Depth | -0.03083 | 0.499 | ≤0.2 | >0.05 |
|  | Shannon | 0.063589 | 0.245 | ≤0.2 | >0.05 |
|  | Salinity | 0.366618 | 0.004 | >0.2 | <0.01 |
|  | Temperature | -0.01887 | 0.51 | ≤0.2 | >0.05 |
|  | pH | -0.08305 | 0.731 | ≤0.2 | >0.05 |
|  | Turbidity | 0.162054 | 0.112 | ≤0.2 | >0.05 |
|  | DO | -0.08507 | 0.711 | ≤0.2 | >0.05 |
|  | DIN | 0.014627 | 0.35 | ≤0.2 | >0.05 |
|  | DSI | 0.197109 | 0.104 | ≤0.2 | >0.05 |
|  | DIP | -0.02292 | 0.537 | ≤0.2 | >0.05 |
| on-PF-PA | Depth | -0.09154 | 0.902 | ≤0.2 | >0.05 |
|  | Shannon | 0.121253 | 0.151 | ≤0.2 | >0.05 |
|  | Salinity | 0.563757 | 0.001 | >0.4 | <0.01 |
|  | Temperature | -0.07245 | 0.797 | ≤0.2 | >0.05 |
|  | pH | -0.16021 | 0.995 | ≤0.2 | >0.05 |
|  | Turbidity | 0.588878 | 0.003 | >0.4 | <0.01 |
|  | DO | -0.16679 | 0.996 | ≤0.2 | >0.05 |
|  | DIN | 0.572977 | 0.003 | >0.4 | <0.01 |
|  | DSI | 0.712161 | 0.002 | >0.6 | <0.01 |
|  | DIP | 0.052991 | 0.222 | ≤0.2 | >0.05 |

*^a^* "on-SF-FL" and "on-SF-PA" individually represent the FL and PA community structure in the water mass of the sediment front, "between-SF-PF-FL" and "between-SF-PF-PA" individually represent the FL and PA community structure in the water mass between the sediment front and the plume front, and "on-PF-FL" and "on-PF-PA" individually represent the FL and PA community structure in the water mass of the plume front.

**Supplementary Table S5.** The value of alpha diversity indices in all seawater samples, including Shannon, Faith_pd, Evenness, and Coverage.

| **Sample-ID** | **Shannon** | **Faith_pd** | **Evenness** | **Coverage** |
| --- | --- | --- | --- | --- |
| YE9-1-0.22 μm | 1222 | 41.56941 | 0.949535 | 0.999905 |
| YE9-2-0.22 μm | 1331 | 55.26598 | 0.94746 | 0.999915 |
| YE10-1-0.22 μm | 1471 | 41.17269 | 0.953236 | 0.999928 |
| YE10-2-0.22 μm | 788 | 29.65954 | 0.915023 | 1 |
| YE11-1-0.22 μm | 1352 | 34.17409 | 0.957893 | 0.999819 |
| YE11-2-0.22 μm | 2499 | 55.23867 | 0.965022 | 1 |
| YE12-1-0.22 μm | 1449 | 36.4967 | 0.962414 | 0.99993 |
| YE12-2-0.22 μm | 1413 | 32.73143 | 0.964956 | 0.99993 |
| YE13-1-0.22 μm | 1467 | 29.90032 | 0.962258 | 0.999885 |
| YE13-2-0.22 μm | 1281 | 31.7718 | 0.950982 | 0.999801 |
| YE14-2-1-0.22 μm | 1405 | 32.8291 | 0.960405 | 0.999903 |
| YE14-2-2-0.22 μm | 1460 | 30.43773 | 0.962178 | 0.999967 |
| YEC7-1-0.22 μm | 1464 | 29.79401 | 0.96415 | 0.999931 |
| YEC7-2-0.22 μm | 1629 | 41.92352 | 0.960393 | 0.999916 |
| YEC7-3-0.22 μm | 1415 | 42.64334 | 0.955835 | 1 |
| YE15-1-0.22 μm | 1607 | 33.08765 | 0.954183 | 0.999876 |
| YE15-2-0.22 μm | 1388 | 32.2143 | 0.959187 | 0.999969 |
| YE15-3-0.22 μm | 1492 | 34.59658 | 0.959803 | 1 |
| EH1-1-0.22 μm | 981 | 20.08075 | 0.916893 | 0.999966 |
| EH1-2-0.22 μm | 1675 | 32.24961 | 0.958441 | 0.999939 |
| EH1-3-0.22 μm | 1344 | 35.06199 | 0.961814 | 1 |
| EH2-1-0.22 μm | 982 | 18.64101 | 0.940565 | 0.999964 |
| EH2-2-0.22 μm | 1446 | 28.42388 | 0.956816 | 0.999967 |
| EH2-3-0.22 μm | 1355 | 27.84943 | 0.954261 | 0.999852 |
| YEC9-1-0.22 μm | 1307 | 23.79567 | 0.960881 | 0.999962 |
| YEC9-2-0.22 μm | 1200 | 36.46861 | 0.951803 | 0.999964 |
| YEC9-3-0.22 μm | 1284 | 45.03088 | 0.953744 | 0.999879 |
| YEC8-1-0.22 μm | 1406 | 11.05225 | 0.965647 | 0.999835 |
| YEC8-2-0.22 μm | 1220 | 20.04585 | 0.970164 | 0.99987 |
| YEC8-3-0.22 μm | 1247 | 35.01203 | 0.950372 | 0.999875 |
| YEC10-1-0.22 μm | 1356 | 19.27389 | 0.96401 | 1 |
| YEC10-2-0.22 μm | 796 | 24.63057 | 0.937609 | 1 |
| YEC10-3-0.22 μm | 799 | 21.36763 | 0.932858 | 0.999958 |
| YE9-S-3 μm | 1354 | 60.76576 | 0.964071 | 1 |
| YE9-D-3 μm | 1479 | 87.87068 | 0.938382 | 1 |
| YE10-S-3 μm | 1541 | 25.15829 | 0.943973 | 1 |
| YE10-D-3 μm | 1392 | 44.5387 | 0.967342 | ? |
| YE11-S-3 μm | 3497 | 103.8209 | 0.963409 | 1 |
| YE11-D-3 μm | 1860 | 72.19333 | 0.958237 | 1 |
| YE12-S-3 μm | 2839 | 56.76947 | 0.967653 | 0.999983 |
| YE12-D-3 μm | 1796 | 42.16089 | 0.955958 | 0.999976 |
| YE13-S-3 μm | 2333 | 79.3235 | 0.95646 | 0.999946 |
| YE13-D-3 μm | 2700 | 59.1724 | 0.961646 | 0.999982 |
| YE14-2-S-3 μm | 1985 | 40.31223 | 0.968965 | 0.999896 |
| YE14-2-D-3 μm | 2558 | 65.0631 | 0.96331 | 0.99989 |
| YEC7-S-3 μm | 2306 | 51.08904 | 0.957197 | 0.99992 |
| YEC7-M-3 μm | 2461 | 69.9509 | 0.963018 | 0.99998 |
| YEC7-D-3 μm | 2774 | 77.23454 | 0.964211 | 0.999801 |
| YE15-S-3 μm | 2142 | 50.87978 | 0.960758 | 0.999898 |
| YE15-M-3 μm | 1521 | 59.1176 | 0.940735 | 1 |
| YE15-D-3 μm | 2016 | 53.29124 | 0.946284 | 1 |
| EH1-S-3 μm | 1268 | 29.21553 | 0.928762 | 0.999975 |
| EH1-M-3 μm | 1431 | 47.80986 | 0.95339 | 0.999706 |
| EH1-D-3 μm | 1769 | 68.21456 | 0.958567 | 0.999975 |
| EH2-S-3 μm | 1509 | 35.09741 | 0.947558 | 0.999886 |
| EH2-M-3 μm | 2243 | 60.59067 | 0.964532 | 1 |
| EH2-D-3 μm | 1517 | 46.44416 | 0.954815 | 0.999913 |
| YEC8-S-3 μm | 2051 | 21.38174 | 0.957735 | 0.999914 |
| YEC8-M-3 μm | 1634 | 25.696 | 0.973584 | 0.999944 |
| YEC8-D-3 μm | 2668 | 75.39776 | 0.960593 | 0.999962 |
| YEC9-S-3 μm | 2173 | 35.753 | 0.961116 | 0.999906 |
| YEC9-M-3 μm | 1848 | 63.22473 | 0.956765 | 1 |
| YEC9-D-3 μm | 2295 | 70.96435 | 0.961924 | 0.999964 |
| YEC10-S-3 μm | 2227 | 35.22045 | 0.949944 | 0.999907 |
| YEC10-M-3 μm | 2063 | 47.33316 | 0.959932 | 0.999923 |
| YEC10-D-3 μm | 1670 | 38.80997 | 0.948425 | 0.999914 |

**Supplementary Table S6.** Overview of the relative abundance of prokaryotes with distinct lifestyle preferences across different water masses at phylum level.

| **Phylum** | **Relative Abundance (%)** | | | | | | | | | | | |
| --- | --- | --- | --- | --- | --- | --- | --- | --- | --- | --- | --- | --- |
|  | **All samples** | **PA** | **FL** | **on-SF** | **between-SF-PF** | **on-PF** | **PA** | | | **FL** | | |
|  |  |  |  |  |  |  | **on-SF** | **between-SF-PF** | **on-PF** | **on-SF** | **between-SF-PF** | **on-PF** |
| *Acetothermia* | 0.0005 | 0.0009 | 0 | 0.0023 | 0 | 0 | 0.0036 | 0 | 0 | 0 | 0 | 0 |
| *Acidobacteriota* | 0.7606 | 1.2245 | 0.0441 | 2.8914 | 0.1545 | 0.0793 | 4.5384 | 0.2386 | 0.1275 | 0.1574 | 0.0153 | 0.0092 |
| *Actinobacteriota* | 8.8315 | 7.3863 | 11.0634 | 12.5166 | 9.3003 | 6.9756 | 15.2995 | 8.4583 | 3.1562 | 7.8972 | 10.6931 | 12.5201 |
| *Aenigmarchaeota* | 0.0003 | 0.0005 | 0 | 0.0005 | 0.0004 | 0.0002 | 0.0008 | 0.0006 | 0.0004 | 0 | 0 | 0 |
| *Aerophobota* | 0.001 | 0.0014 | 0.0003 | 0.0035 | 0.0005 | 0 | 0.0056 | 0 | 0 | 0 | 0.0014 | 0 |
| *Altiarchaeota* | 0.0003 | 0.0005 | 0 | 0 | 0 | 0.0006 | 0 | 0 | 0.0011 | 0 | 0 | 0 |
| *AncK6* | 0.0002 | 0.0003 | 0 | 0.0005 | 0 | 0.0002 | 0.0008 | 0 | 0.0003 | 0 | 0 | 0 |
| *Armatimonadota* | 0.0228 | 0.0358 | 0.0027 | 0.0786 | 0.0116 | 0.0029 | 0.1235 | 0.0163 | 0.0037 | 0.0042 | 0.0037 | 0.0017 |
| *Asgardarchaeota* | 0.0005 | 0.0005 | 0.0005 | 0.0007 | 0.0005 | 0.0004 | 0.0011 | 0.0008 | 0 | 0 | 0 | 0.001 |
| *Bacteroidota* | 14.1781 | 12.8547 | 16.222 | 8.6981 | 17.7124 | 15.0479 | 6.9318 | 16.8111 | 13.7838 | 11.6301 | 19.203 | 16.8829 |
| *Bdellovibrionota* | 0.6806 | 0.95 | 0.2644 | 0.55 | 0.3903 | 0.8686 | 0.7254 | 0.5141 | 1.2607 | 0.259 | 0.1855 | 0.2992 |
| *Calditrichota* | 0.008 | 0.0132 | 0 | 0.0277 | 0.0014 | 0.0022 | 0.0443 | 0.0022 | 0.0037 | 0 | 0 | 0 |
| *Campilobacterota* | 0.2066 | 0.3316 | 0.0135 | 0.8206 | 0.0272 | 0.0124 | 1.297 | 0.0371 | 0.0155 | 0.0298 | 0.0107 | 0.0079 |
| *Chloroflexi* | 0.2218 | 0.3308 | 0.0534 | 0.657 | 0.1533 | 0.0579 | 1.0068 | 0.2206 | 0.0642 | 0.0764 | 0.0418 | 0.0487 |
| *CK-2C2-2* | 0.0004 | 0.0005 | 0.0003 | 0.0009 | 0.0005 | 0.0002 | 0.0014 | 0 | 0.0003 | 0 | 0.0014 | 0 |
| *Crenarchaeota* | 3.6507 | 2.985 | 4.6788 | 2.9197 | 5.5973 | 3.1078 | 1.8355 | 5.9191 | 2.1469 | 4.7195 | 5.065 | 4.5027 |
| *Cyanobacteria* | 5.9311 | 4.3109 | 8.4333 | 1.53 | 7.0601 | 7.3931 | 0.96 | 8.3787 | 3.9764 | 2.4762 | 4.8791 | 12.353 |
| *Dadabacteria* | 0.0241 | 0.026 | 0.0211 | 0.0308 | 0.0335 | 0.0169 | 0.0424 | 0.0478 | 0.0081 | 0.0116 | 0.0098 | 0.0297 |
| *Deferrisomatota* | 0.0047 | 0.0075 | 0.0003 | 0.0144 | 0.0018 | 0.0016 | 0.0222 | 0.0028 | 0.0028 | 0.0014 | 0 | 0 |
| *Deinococcota* | 0.021 | 0.034 | 0.0007 | 0.0827 | 0.0054 | 0.0003 | 0.1305 | 0.0087 | 0.0005 | 0.0033 | 0 | 0 |
| *Dependentiae* | 0.0035 | 0.004 | 0.0027 | 0.0098 | 0.0005 | 0.002 | 0.0104 | 0 | 0.0029 | 0.0089 | 0.0014 | 0.0008 |
| *Desulfobacterota* | 0.6955 | 1.0658 | 0.1236 | 0.7926 | 0.7849 | 0.6122 | 1.1971 | 1.1195 | 0.9786 | 0.1211 | 0.2315 | 0.0802 |
| *DTB120* | 0.0002 | 0.0004 | 0 | 0.0005 | 0.0005 | 0 | 0.0008 | 0.0008 | 0 | 0 | 0 | 0 |
| *Elusimicrobiota* | 0.0067 | 0.0066 | 0.0069 | 0.018 | 0.0035 | 0.0031 | 0.0224 | 0.0039 | 0.0004 | 0.0107 | 0.0028 | 0.0071 |
| *Entotheonellaeota* | 0.0004 | 0.0007 | 0 | 0.0018 | 0 | 0 | 0.0028 | 0 | 0 | 0 | 0 | 0 |
| *Euryarchaeota* | 0.001 | 0.0016 | 0 | 0.0039 | 0.0004 | 0 | 0.0062 | 0.0006 | 0 | 0 | 0 | 0 |
| *Fibrobacterota* | 0.0062 | 0.008 | 0.0034 | 0.0047 | 0.0054 | 0.0071 | 0.0056 | 0.0059 | 0.01 | 0.0033 | 0.0046 | 0.0029 |
| *Firmicutes* | 0.2606 | 0.339 | 0.1395 | 0.179 | 0.2174 | 0.3163 | 0.2366 | 0.1897 | 0.4573 | 0.0834 | 0.2631 | 0.1116 |
| *Fusobacteriota* | 0.0013 | 0.0019 | 0.0004 | 0.0023 | 0.0011 | 0.001 | 0.0025 | 0.0017 | 0.0017 | 0.0019 | 0 | 0 |
| *GAL15* | 0.0001 | 0.0002 | 0 | 0.0005 | 0 | 0 | 0.0008 | 0 | 0 | 0 | 0 | 0 |
| *Gemmatimonadota* | 0.2036 | 0.3017 | 0.052 | 0.4303 | 0.1638 | 0.1201 | 0.6435 | 0.224 | 0.1773 | 0.0764 | 0.0642 | 0.037 |
| *GN01* | 0.0001 | 0.0001 | 0 | 0 | 0.0004 | 0 | 0 | 0.0006 | 0 | 0 | 0 | 0 |
| *Halobacterota* | 0.001 | 0.0016 | 0 | 0.0026 | 0.0004 | 0.0005 | 0.0042 | 0.0006 | 0.0008 | 0 | 0 | 0 |
| *Hydrogenedentes* | 0.0072 | 0.0112 | 0.0011 | 0.0158 | 0.004 | 0.0048 | 0.0241 | 0.0059 | 0.0075 | 0.0019 | 0.0009 | 0.0008 |
| *Hydrothermarchaeota* | 0.0003 | 0.0005 | 0 | 0 | 0.0012 | 0 | 0 | 0.002 | 0 | 0 | 0 | 0 |
| *Iainarchaeota* | 0.0004 | 0.0004 | 0.0004 | 0.0011 | 0 | 0.0003 | 0.0017 | 0 | 0 | 0 | 0 | 0.0008 |
| *Latescibacterota* | 0.0367 | 0.0579 | 0.0039 | 0.1091 | 0.0259 | 0.0092 | 0.1664 | 0.0416 | 0.0145 | 0.014 | 0 | 0.0013 |
| *LCP-89* | 0.0002 | 0.0004 | 0 | 0 | 0 | 0.0005 | 0 | 0 | 0.0008 | 0 | 0 | 0 |
| *Margulisbacteria* | 0.013 | 0.0114 | 0.0156 | 0.0102 | 0.027 | 0.0081 | 0.0095 | 0.0259 | 0.0054 | 0.0112 | 0.0288 | 0.0119 |
| *Marinimicrobia*  *(SAR406_clade)* | 0.4347 | 0.2778 | 0.6772 | 0.2364 | 0.5886 | 0.4547 | 0.1675 | 0.502 | 0.2242 | 0.3507 | 0.7317 | 0.7891 |
| *MBNT15* | 0.0237 | 0.0369 | 0.0033 | 0.0804 | 0.014 | 0.0027 | 0.1201 | 0.0225 | 0.0045 | 0.0144 | 0 | 0 |
| *Methylomirabilota* | 0.0007 | 0.0012 | 0 | 0.0026 | 0.0004 | 0 | 0.0042 | 0.0006 | 0 | 0 | 0 | 0 |
| *Modulibacteria* | 0.0005 | 0.0008 | 0 | 0.0014 | 0.0004 | 0.0002 | 0.0022 | 0.0006 | 0.0003 | 0 | 0 | 0 |
| *Myxococcota* | 0.1972 | 0.2968 | 0.0433 | 0.4656 | 0.1293 | 0.1076 | 0.7139 | 0.1765 | 0.157 | 0.0536 | 0.0511 | 0.0359 |
| *Nanoarchaeota* | 0.1031 | 0.0963 | 0.1137 | 0.1417 | 0.1447 | 0.0673 | 0.1381 | 0.1462 | 0.0531 | 0.1477 | 0.1423 | 0.0879 |
| *NB1-j* | 0.0838 | 0.1331 | 0.0078 | 0.2261 | 0.0345 | 0.0423 | 0.3505 | 0.0526 | 0.0686 | 0.0196 | 0.0046 | 0.0042 |
| *Nitrospinota* | 0.0434 | 0.0441 | 0.0424 | 0.025 | 0.0751 | 0.0375 | 0.0337 | 0.0936 | 0.0258 | 0.0107 | 0.0446 | 0.0545 |
| *Nitrospirota* | 0.0235 | 0.0365 | 0.0035 | 0.0806 | 0.0112 | 0.0035 | 0.1218 | 0.0169 | 0.0055 | 0.0121 | 0.0019 | 0.0006 |
| *NKB15* | 0.004 | 0.0066 | 0 | 0.0158 | 0 | 0.0005 | 0.0253 | 0 | 0.0009 | 0 | 0 | 0 |
| *Patescibacteria* | 0.1379 | 0.1975 | 0.0459 | 0.2513 | 0.0534 | 0.125 | 0.3802 | 0.0542 | 0.1788 | 0.0373 | 0.0521 | 0.047 |
| *PAUC34f* | 0.0556 | 0.0586 | 0.051 | 0.0823 | 0.086 | 0.0301 | 0.0926 | 0.097 | 0.0246 | 0.0652 | 0.0679 | 0.0382 |
| *Planctomycetota* | 3.7966 | 5.634 | 0.959 | 2.4047 | 3.5538 | 4.5269 | 3.5346 | 5.0832 | 6.8815 | 0.5291 | 1.0242 | 1.1091 |
| *Poribacteria* | 0.0004 | 0.0007 | 0 | 0 | 0.0004 | 0.0006 | 0 | 0.0006 | 0.0011 | 0 | 0 | 0 |
| *Proteobacteria* | 48.2717 | 45.4945 | 52.5609 | 58.3376 | 46.0756 | 44.7554 | 52.6203 | 42.6281 | 43.4866 | 67.8281 | 51.7778 | 46.5971 |
| *RCP2-54* | 0.0589 | 0.0888 | 0.0127 | 0.0877 | 0.0596 | 0.0458 | 0.1333 | 0.0767 | 0.0736 | 0.0121 | 0.0311 | 0.0054 |
| *SAR324_clade*  *(Marine_group_B)* | 0.7142 | 0.5713 | 0.935 | 0.7773 | 1.1405 | 0.4957 | 0.8289 | 0.9863 | 0.2549 | 0.6917 | 1.3956 | 0.8451 |
| *Schekmanbacteria* | 0.0085 | 0.014 | 0 | 0.0303 | 0.004 | 0.0007 | 0.0485 | 0.0065 | 0.0012 | 0 | 0 | 0 |
| *Spirochaetota* | 0.0142 | 0.0177 | 0.0087 | 0.0366 | 0.0019 | 0.0096 | 0.0497 | 0.0008 | 0.0106 | 0.0149 | 0.0037 | 0.0082 |
| *Sumerlaeota* | 0.0026 | 0.004 | 0.0003 | 0.0063 | 0.0042 | 0.0002 | 0.0101 | 0.0059 | 0.0003 | 0 | 0.0014 | 0 |
| *Sva0485* | 0.0062 | 0.0095 | 0.0011 | 0.0179 | 0.0035 | 0.0021 | 0.0258 | 0.0056 | 0.0036 | 0.0047 | 0 | 0 |
| *TA06* | 0.0001 | 0.0002 | 0 | 0 | 0.0005 | 0 | 0 | 0.0008 | 0 | 0 | 0 | 0 |
| *Thermoplasmatota* | 1.2097 | 0.9378 | 1.6296 | 0.6973 | 2.5406 | 0.8441 | 0.3511 | 2.6623 | 0.4034 | 1.2721 | 2.3394 | 1.4839 |
| *TX1A-33* | 0.0001 | 0 | 0.0003 | 0.0005 | 0 | 0 | 0 | 0 | 0 | 0.0014 | 0 | 0 |
| *Verrucomicrobiota* | 1.0591 | 1.1641 | 0.8969 | 0.7751 | 1.2188 | 1.1147 | 0.9025 | 1.3935 | 1.1795 | 0.5636 | 0.9298 | 1.0207 |
| *WPS-2* | 0.0016 | 0.001 | 0.0025 | 0.0039 | 0.0009 | 0.0009 | 0.0031 | 0.0008 | 0 | 0.0051 | 0.0009 | 0.0021 |
| *WS1* | 0.0012 | 0.002 | 0 | 0.0044 | 0 | 0.0004 | 0.007 | 0 | 0.0007 | 0 | 0 | 0 |
| *WS2* | 0.0067 | 0.011 | 0.0002 | 0.0261 | 0.0025 | 0 | 0.0413 | 0.0039 | 0 | 0.0009 | 0 | 0 |
| *WS4* | 0.0001 | 0.0002 | 0 | 0.0005 | 0 | 0 | 0.0008 | 0 | 0 | 0 | 0 | 0 |
| *Zixibacteria* | 0.0095 | 0.0153 | 0.0004 | 0.0235 | 0.0109 | 0.0026 | 0.037 | 0.0169 | 0.0044 | 0.0009 | 0.0009 | 0 |
| Others | 7.9475 | 12.5395 | 0.8557 | 2.753 | 2.5578 | 12.6758 | 3.9505 | 3.686 | 20.7462 | 0.7653 | 0.6918 | 0.9606 |

*^a^* “Others” represents the uncultured and unknown families.

**Supplementary Table S7.** Overview of the relative abundance of prokaryotes with distinct lifestyle preferences across different water masses at family level.

| **Family** | **Relative Abundance (%)** | | | | | | | | | | | |
| --- | --- | --- | --- | --- | --- | --- | --- | --- | --- | --- | --- | --- |
|  | **All samples** | **PA** | **FL** | **on-SF** | **between-SF-PF** | **on-PF** | **PA** | | | **FL** | | |
|  |  |  |  |  |  |  | **on-SF** | **between-SF-PF** | **on-PF** | **on-SF** | **between-SF-PF** | **on-PF** |
| *Rhodobacteraceae* | 15.5769 | 15.4925 | 15.692 | 12.5345 | 12.4584 | 18.645 | 9.898 | 10.3488 | 21.6239 | 16.7705 | 15.8426 | 15.1847 |
| *Flavobacteriaceae* | 8.5249 | 6.384 | 11.4415 | 4.4538 | 11.0825 | 9.2757 | 3.0519 | 9.965 | 6.2397 | 6.7062 | 12.8751 | 12.8023 |
| *Cyanobiaceae* | 6.3903 | 4.8393 | 8.5033 | 1.5523 | 7.2364 | 8.3757 | 0.9688 | 8.6877 | 4.8495 | 2.4897 | 4.9084 | 12.4718 |
| *Actinomarinaceae* | 5.7308 | 3.0676 | 9.3591 | 4.6132 | 6.5025 | 5.9022 | 3.3005 | 5.2027 | 1.7148 | 6.7222 | 8.5875 | 10.7663 |
| *Nitrosopumilaceae* | 3.96 | 3.4031 | 4.7188 | 2.9954 | 5.7334 | 3.5561 | 1.9002 | 6.1281 | 2.7039 | 4.755 | 5.1003 | 4.546 |
| *SAR86_clade* | 3.8197 | 2.5311 | 5.5752 | 3.9545 | 5.314 | 3.0078 | 2.5938 | 4.4883 | 1.3772 | 6.1406 | 6.6386 | 4.902 |
| *Halieaceae* | 3.7313 | 3.8423 | 3.5799 | 5.7779 | 4.3384 | 2.4103 | 7.7793 | 4.1548 | 1.417 | 2.5624 | 4.6331 | 3.564 |
| *SAR11_Clade_I* | 3.6478 | 2.6343 | 5.0285 | 2.7802 | 5.5763 | 3.1182 | 1.6577 | 5.0139 | 1.832 | 4.5836 | 6.4785 | 4.6122 |
| *Stappiaceae* | 3.2368 | 4.3919 | 1.6631 | 1.3882 | 3.7059 | 3.9229 | 1.7512 | 4.6739 | 5.7379 | 0.805 | 2.1529 | 1.8146 |
| *Saprospiraceae* | 2.8152 | 4.1574 | 0.9867 | 1.2522 | 2.8237 | 3.5887 | 1.3895 | 3.5337 | 6.0933 | 1.0317 | 1.6848 | 0.6792 |
| *Pirellulaceae* | 2.6961 | 4.2229 | 0.616 | 1.4754 | 2.4583 | 3.4221 | 2.1842 | 3.5402 | 5.7765 | 0.3366 | 0.7228 | 0.6872 |
| *Alteromonadaceae* | 1.807 | 0.7328 | 3.2704 | 5.1937 | 0.3771 | 0.8345 | 1.1318 | 0.1199 | 0.8553 | 11.7198 | 0.7897 | 0.8103 |
| *AEGEAN-169_marine_group* | 1.6889 | 1.1093 | 2.4784 | 1.7177 | 2.4946 | 1.2729 | 1.0903 | 2.0454 | 0.5854 | 2.7258 | 3.2151 | 2.0716 |
| *Microtrichaceae* | 1.6878 | 1.9041 | 1.3931 | 1.7336 | 1.8839 | 1.5673 | 2.3449 | 2.0764 | 1.5541 | 0.7515 | 1.5753 | 1.5825 |
| *Nitrincolaceae* | 1.3209 | 2.1864 | 0.1417 | 4.8525 | 0.1454 | 0.1495 | 7.7065 | 0.1637 | 0.1917 | 0.2671 | 0.1161 | 0.1005 |
| *Oleiphilaceae* | 1.3083 | 1.9002 | 0.502 | 0.0306 | 0.1968 | 2.4981 | 0.0085 | 0.2486 | 3.9233 | 0.0662 | 0.1138 | 0.8425 |
| *Marine_Group_II* | 1.285 | 1.0424 | 1.6155 | 0.7153 | 2.5784 | 0.9238 | 0.364 | 2.7306 | 0.465 | 1.2796 | 2.3341 | 1.4568 |
| *Vibrionaceae* | 1.2531 | 1.495 | 0.9236 | 0.7486 | 0.343 | 1.9578 | 0.2174 | 0.4395 | 2.8272 | 1.602 | 0.1882 | 0.9479 |
| *UBA10353_marine_group* | 1.1345 | 1.2971 | 0.913 | 1.9623 | 1.2289 | 0.6756 | 2.651 | 1.3719 | 0.4817 | 0.8557 | 0.9995 | 0.9008 |
| *Crocinitomicaceae* | 1.0922 | 1.1643 | 0.9941 | 0.6461 | 1.7782 | 0.9723 | 0.4479 | 1.7203 | 1.2555 | 0.9646 | 1.8711 | 0.6434 |
| *PeM15* | 0.9908 | 1.6616 | 0.077 | 3.5352 | 0.2731 | 0.0826 | 5.7234 | 0.3557 | 0.0897 | 0.0197 | 0.1404 | 0.0744 |
| *Hyphomonadaceae* | 0.92 | 1.4318 | 0.2227 | 0.0153 | 0.2982 | 1.6801 | 0.0085 | 0.3706 | 2.8504 | 0.0263 | 0.1821 | 0.3205 |
| *NS9_marine_group* | 0.8902 | 0.9199 | 0.8496 | 0.486 | 0.6693 | 1.2013 | 0.4759 | 0.6557 | 1.3242 | 0.5023 | 0.691 | 1.0586 |
| *Pseudoalteromonadaceae* | 0.86 | 0.9447 | 0.7446 | 0.7331 | 0.1821 | 1.261 | 0.201 | 0.1926 | 1.7989 | 1.588 | 0.1653 | 0.6362 |
| *Cryomorphaceae* | 0.809 | 0.6291 | 1.054 | 1.3052 | 0.8708 | 0.5312 | 0.9597 | 0.6662 | 0.4192 | 1.8602 | 1.1989 | 0.6614 |
| *SAR324_clade*  *(Marine_group_B)* | 0.7759 | 0.6532 | 0.9431 | 0.7994 | 1.1704 | 0.5676 | 0.863 | 1.024 | 0.3216 | 0.697 | 1.4053 | 0.8533 |
| *Thioglobaceae* | 0.7534 | 0.5096 | 1.0856 | 0.1439 | 0.9569 | 0.9553 | 0.1099 | 0.9534 | 0.4842 | 0.1986 | 0.9625 | 1.5025 |
| *Sphingomonadaceae* | 0.7151 | 0.4546 | 1.0701 | 1.4966 | 0.2501 | 0.5581 | 0.719 | 0.218 | 0.4388 | 2.746 | 0.3015 | 0.6967 |
| *Moraxellaceae* | 0.5844 | 0.055 | 1.3057 | 1.851 | 0.0025 | 0.2442 | 0.1548 | 0.0015 | 0.0285 | 4.5761 | 0.0042 | 0.4947 |
| *SAR116_clade* | 0.574 | 0.3554 | 0.8719 | 0.4524 | 0.8276 | 0.5082 | 0.3602 | 0.5504 | 0.2413 | 0.6004 | 1.2724 | 0.8183 |
| *Woeseiaceae* | 0.571 | 0.9061 | 0.1145 | 1.7521 | 0.2332 | 0.1518 | 2.7437 | 0.3566 | 0.1714 | 0.1591 | 0.0351 | 0.129 |
| *SAR11_Clade_II* | 0.5656 | 0.3893 | 0.8057 | 0.2409 | 0.8643 | 0.5782 | 0.1581 | 0.6928 | 0.3478 | 0.3741 | 1.1394 | 0.8458 |
| *Phycisphaeraceae* | 0.5471 | 0.8233 | 0.1707 | 0.1019 | 0.1541 | 0.9644 | 0.1385 | 0.1949 | 1.5731 | 0.0432 | 0.0885 | 0.2574 |
| *Bdellovibrionaceae* | 0.5323 | 0.7829 | 0.191 | 0.3297 | 0.3344 | 0.7318 | 0.4447 | 0.4488 | 1.1668 | 0.145 | 0.1507 | 0.2266 |
| *Pseudomonadaceae* | 0.4946 | 0.1796 | 0.9239 | 1.5975 | 0.1359 | 0.1247 | 0.6281 | 0.0374 | 0.0048 | 3.1548 | 0.294 | 0.264 |
| *OM182_clade* | 0.4781 | 0.3996 | 0.585 | 0.6722 | 0.6369 | 0.3023 | 0.5601 | 0.6537 | 0.1629 | 0.8524 | 0.61 | 0.4642 |
| *Marinimicrobia_(SAR406_clade)* | 0.4723 | 0.3176 | 0.683 | 0.2431 | 0.604 | 0.5207 | 0.1744 | 0.5212 | 0.2829 | 0.3535 | 0.7368 | 0.7968 |
| *Rubritaleaceae* | 0.4417 | 0.5358 | 0.3136 | 0.1889 | 0.2306 | 0.6727 | 0.1952 | 0.2206 | 0.9102 | 0.1788 | 0.2467 | 0.3968 |
| *Gimesiaceae* | 0.3993 | 0.6406 | 0.0706 | 0.3774 | 0.7158 | 0.2525 | 0.5604 | 1.0672 | 0.4427 | 0.0836 | 0.1521 | 0.0316 |
| *DEV007* | 0.3079 | 0.3527 | 0.2468 | 0.1313 | 0.4771 | 0.3114 | 0.1356 | 0.5854 | 0.3436 | 0.1244 | 0.3034 | 0.2739 |
| *Ilumatobacteraceae* | 0.2828 | 0.3974 | 0.1268 | 0.7129 | 0.2323 | 0.0941 | 1.0854 | 0.2714 | 0.0767 | 0.1145 | 0.1695 | 0.1143 |
| *Saccharospirillaceae* | 0.2783 | 0.4009 | 0.1111 | 0.089 | 0.0422 | 0.4901 | 0.0783 | 0.0587 | 0.7806 | 0.1061 | 0.0159 | 0.1526 |
| *Alcanivoracaceae1* | 0.2759 | 0.1006 | 0.5148 | 0.2996 | 0.3453 | 0.2296 | 0.1969 | 0.1389 | 0.0238 | 0.4647 | 0.6765 | 0.4685 |
| *Cyclobacteriaceae* | 0.2561 | 0.2074 | 0.3224 | 0.2143 | 0.153 | 0.3283 | 0.2375 | 0.1494 | 0.2234 | 0.177 | 0.1587 | 0.4501 |
| *OM190* | 0.2502 | 0.3997 | 0.0466 | 0.1603 | 0.1972 | 0.3214 | 0.239 | 0.2936 | 0.552 | 0.0338 | 0.0426 | 0.0535 |
| *Pseudohongiellaceae* | 0.2452 | 0.1677 | 0.3508 | 0.2469 | 0.3245 | 0.2048 | 0.2311 | 0.2682 | 0.074 | 0.2722 | 0.4148 | 0.3568 |
| Others | 4.6618 | 6.868 | 1.656 | 6.7895 | 4.3081 | 3.7793 | 9.9234 | 5.4102 | 5.9571 | 1.7546 | 2.5401 | 1.2496 |

*^a^* “Others” represents the families excluded from the cumulative 90% relative abundance in all samples, uncultured and unknown families.
